# Performance, feed utilization, and hepatic metabolic response of weaned juvenile Atlantic bluefin tuna (*Thunnus thynnus* L.): effects of dietary lipid level and source

**DOI:** 10.1007/s10695-018-0587-9

**Published:** 2018-11-23

**Authors:** Mónica B. Betancor, Aurelio Ortega, Fernando de la Gándara, Douglas R. Tocher, Gabriel Mourente

**Affiliations:** 1 Institute of Aquaculture, Faculty of Natural Sciences, University of Stirling, Stirling, Scotland FK9 4LA UK; 2 Planta Experimental de Cultivos Marinos, Instituto Español de Oceanografía (IEO), 30860 Puerto de Mazarrón, Murcia, Spain; 3 Departamento de Biología, Facultad de Ciencias del Mar y Ambientales, Universidad de Cádiz, 11510 Puerto Real, Cádiz, Spain

**Keywords:** Atlantic bluefin tuna, Dietary lipid content, Dietary lipid source, Production performance, Hepatic lipid metabolism, Gene expression

## Abstract

Two trials were performed using extruded diets as on-growing feeds for weaned Atlantic bluefin tuna (*Thunnus thynnus*; ABT) to establish adequate dietary levels of both lipid and omega-3 long-chain polyunsaturated fatty acids (LC-PUFAs), and impacts on lipid metabolism via liver gene expression. In trial A, ABT were fed with either a commercial feed (Magokoro®; MGK) as a reference diet or two experimental feeds differing in lipid levels (15 or 20%) using krill oil (KO) as the single lipid source in order to estimate suitable lipid content. Fish fed MGK displayed the highest growth, followed by 15KO, and therefore a dietary lipid content of 15% was considered preferable to 20% at this stage. In trial B, fish were fed MGK, 15KO, or a feed containing 15% lipid with a blend of KO and rapeseed oil (RO) (1:1, *v*/*v*; 15KORO). Fish fed 15KO and 15KORO showed no difference in weight gain, specific growth rate, and fork length. Increasing dietary lipid level or including vegetable oil, RO, in the feeds did not increase liver lipid content. Liver fatty acid compositions largely reflected dietary profiles confirming very limited endogenous LC-PUFA biosynthesis. Liver of ABT fed 15KO and 20KO displayed the highest contents of docosahexaenoic acid (DHA). The hepatic expression of genes encoding enzymes and transcription factors involved in lipid and fatty acid metabolism, as well as genes encoding antioxidant enzymes, showed that many of these genes were regulated by dietary lipid and LC-PUFA content. Results suggested that ABT juveniles can be on-grown on inert dry feeds that support good fish growth and the accumulation of DHA.

**Electronic supplementary material:**

The online version of this article (10.1007/s10695-018-0587-9) contains supplementary material, which is available to authorized users.

## Introduction

Currently, the vast majority of Atlantic bluefin tuna (ABT) production from aquaculture is derived from fattening operations based in the Mediterranean Sea with the three main producer countries being Spain, Croatia, and Malta (Benetti et al. 2016; van Beijnen 2017). Essentially, captured wild ABT (mostly adult broodstock fish and some juveniles) are towed in sea pens to fattening farms where they are fed mainly on baitfish of variable quality (Benetti et al. 2016; van Beijnen 2017). The development of formulated sustainable feed is essential for the farming of tuna species, whether in fattening or, ultimately, closed life-cycle culture (van Beijnen 2017). The advantages of formulated diets include choice of the most nutritionally adequate ingredients, range of pellet size and consistent nutritional content, and reduced need for live feeds for larvae, and baitfish for hatchery-reared juveniles from 4 weeks after hatch onwards and, thus, the prevention of pathogen transmission. The development of artificial diets for Pacific bluefin tuna (*Thunnus orientalis*) (PBT) has been carried out extensively in Japan since 1995 (Kenji 2012) but, until recently, similar research has not been possible with Atlantic bluefin tuna (ABT; *Thunnus thynnus* L.) (Mourente and Tocher 2003, 2009). Biswas et al. (2009) concluded that a formulated diet with 62% crude protein and 18% crude lipid could ensure good growth in PBT juveniles. Subsequently, formulated diets for rearing juvenile PBT, including nutrient sources and requirements for crude protein, lipids, carbohydrates, and vitamin C, were established (Biswas 2010; Biswas et al. 2016). Moreover, the partial or full replacement of fish oil (FO) sources with soybean oil (SO) for this species was also investigated (Biswas et al. 2011).

It is well known that lipid is required in the diet of fish to supply both metabolic energy and key nutrients such as essential fatty acids (EFAs) (Sargent et al. 2002). Appropriate uptake, assimilation, and accumulation of lipids improves growth and survival of all fish, but is particularly important in highly active migratory predator fish species such as tunas (Mourente and Tocher 2003, 2009). Additionally, omega-3 (n−3) long-chain polyunsaturated fatty acids (LC-PUFAs; ≥ 20 carbons and ≥ 3 double bonds), such as eicosapentaenoic acid (EPA; 20:5n−3) and docosahexaenoic acid (DHA; 22:6n−3), are required by most marine fish and are EFA for survival, normal growth, and development (Tocher 2010). The tendency nowadays regarding the formulation of aquafeeds is the replacement of marine ingredients with more sustainable raw materials such as plant proteins and vegetable oils of agricultural origin. Such terrestrial ingredients are devoid of EPA and DHA, making fish fed sustainable feeds dependent on endogenous production of n−3 LC-PUFA via metabolic biosynthesis pathways and the expression of key enzymes including fatty acyl desaturases (Fads) and elongases of very long-chain fatty acids (Elovl) (Tocher 2010; Betancor et al. 2015a). However, the capacity for the biosynthesis of EPA and DHA is very limited in bluefin tuna species (Gregory et al. 2010; Morais et al. 2011; Scholefield et al. 2015) and thus the response of these species when fed sustainable feeds with reduced levels of marine ingredients is unknown.

Studies have shown that dietary lipid content can have significant effects on gene expression in salmonids (Martinez-Rubio et al. 2013; Librán-Pérez et al. 2015; Hixson et al. 2017). Specifically, dietary lipid content affects the expression of key genes involved in the major lipid metabolic pathways in liver, as well the major transcription factors and nuclear receptors controlling and regulating the expression of these genes. This all impacts the lipid and fatty acid composition of liver (Martinez-Rubio et al. 2013). Furthermore, recent studies have shown that not only dietary lipid content but also LC-PUFA content can significantly affect the expression of a range of genes associated with lipid metabolism, as might be expected, and other pathways including antioxidant genes (Betancor et al. 2014a; Glencross et al. 2015). Thus, studying the metabolic impact of dietary lipid, including effects on lipid and fatty acid compositions, and the expression of genes of major lipid metabolic pathways is highly relevant in ABT. In addition, the high culture temperature conditions (~ 28 °C), strong aeration/oxygenation, and the high level of dietary pro-oxidants used for rearing ABT may promote highly pro-oxidative conditions. In consequence, it is of considerable relevance to evaluate the antioxidant protection status of juvenile ABT.

The overarching aim of the present study was to determine the impacts of dietary lipid content (15% vs. 20%; trial A) and dietary lipid source (100% krill oil vs. 50% krill oil/50% rapeseed oil; trial B) on fish performance, feed utilization, and expression of genes of lipid metabolism and regulation in liver, and the antioxidant system in ABT juveniles recently weaned from live feeds to inert formulated feed. In order to do so, extruded experimental feeds based on dried fish hydrolysate, fishmeal, and squid meal as protein source plus krill oil and rapeseed oil as lipid sources were formulated, produced, and fed to weaned juvenile ABT (41 days after hatch, dah).

## Materials and methods

### Experimental fish

ABT juveniles were produced from two different batches of eggs spawned in summer 2017 from captive wild broodstock fish maintained in floating net cages located at El Gorguel, off the Cartagena coast, SE Spain. The collected eggs were transferred to the Planta Experimental de Cultivos Marinos, Instituto Español de Oceanografía (IEO), Puerto de Mazarrón (Murcia), Spain for hatching and initial larviculture (Ortega 2015; De la Gándara et al. 2016). Fish were weaned from the live feed stage, fed gilthead sea bream (*Sparus aurata* L.) yolk sac larvae as prey, to formulated feed at 27 dah, using a commercial diet (Magokoro®; MGK; Marubeni Nisshin Feed Co., Japan), which had been successfully used as a formulated feed for PBT (Okada et al. 2014; Kurata et al. 2015; Honryo et al. 2018). The ABT were weaned using MGK at 0.6 to 0.9 mm pellet sizes and were completely weaned by 32 dah.

### Experimental diets

The nutritional trials consisted of two consecutive 10-day feeding trials (A and B) with weaned 41 dah ABT juveniles, from two different batches of spawned ABT eggs. Each trial investigated two experimental extruded feeds in comparison to the commercial reference feed (MGK), which was used in the present trials to benchmark the experimental feeds. Trial A tested dietary lipid level and used two experimental feeds formulated with krill oil (KO) to supply lipid at 15% and 20% on a dry mass basis (15KO and 20KO, respectively) in comparison to the commercial MGK reference feed, which was formulated with fish oil (FO). Trial B examined the effects of replacing 50% of KO with rapeseed oil (RO), to determine the impact of different dietary fatty acid composition, using two experimental feeds formulated to supply 15% lipid, one with 100% KO (15KO), the other with 50% KO and 50% RO (15KORO), in comparison to the MGK reference. The three experimental diets (15KO, 20KO, and 15KORO) were formulated and produced by extrusion (Sparos Lda., Olhão, Portugal). The formulations and analyzed proximate compositions of the reference and test diets are shown in Tables 1 and 2. Crude protein was approximately 56% on a dry-matter basis and crude lipid content was 17.9% in the reference diet, 14.3% and 14.7% in the test diets 15KO and 15KORO, respectively, while diet 20KO contained 18.9% total lipid. Diets 15KO and 15KORO were isocaloric (20.2 kJ g^−1^) whereas diet 20KO had higher energy level (21.3 kJ g^−1^).

**Table 1:** Formulation of the experimental feeds

	15KO^a^	20KO^b^	15KORO^c^
Ingredients (%)			
Micronized fish meal^1^	12.0	12.6	12.5
Squid meal^2^	15.0	15.7	15.0
CPSP 90^3^	30.0	30.0	30.0
Krill oil^4^	9.4	13.9	4.7
Rapeseed oil^5^	–	–	4.7
Algatrium DHA 70^6^	2.5	3.5	2.0
Starch^7^	9.8	3.0	9.8
Vitamin and mineral premix^8^	2.00	2.00	2.00
Lutavit C 35^9^	0.10	0.10	0.10
NaH_2_PO_4_^10^	4.50	4.50	4.50
Taurine^11^	2.00	2.00	2.00
Attractant AA mix^12^	0.50	0.50	0.50
Technical additives^13^	2.70	2.70	2.70
Organic Se^14^	0.50	0.50	0.50
Binders^15^	9.00	9.00	9.00

^a^15KO, 15% total lipid as krill oil

^b^20KO, 20% total lipid as krill oil

^c^15KORO, 15 total lipid as 50% krill oil and 50% European rapeseed oil

^1^Micronorse, Tromsø Fiskeindustri A/S, Tromsø, Norway

^2^Sopropêche, France

^3^CPSP 90, fresh fish by-products enzymatically hydrolyzed soluble fish protein concentrated (82–86% protein), Sopropêche, France

^4^Qrill Aqua phospholipid oil, Aker Biomarine, Norway

^5^Euroingredientes, Cacém, Portugal

^6^Neoquimica, Carregado, Portugal

^7^Formulab, Maia, Portugal

^8^Vitamin and mineral premix. Vitamins (IU or mg kg^−1^ diet): sodium menadione bisulfate, 40 mg; retinyl acetate, 32,000 IU; dl-cholecalciferol, 6800 IU; thiamin, 32 mg; riboflavin, 80 mg; pyridoxine, 40 mg; cyanocobalamin, 0.08 mg; nicotinic acid, 280 mg; folic acid, 24 mg; inositol, 1200 mg; biotin, 2.8 mg; calcium panthotenate, 120 mg; betaine, 1600 mg. Minerals (g or mg/kg diet): cobalt carbonate, 1.3 mg; copper sulfate, 18 mg; ferric sulfate, 12 mg; potassium iodide, 1.0 mg; manganese oxide, 19.2 mg; sodium selenite, 0.02 mg; zinc sulfate, 15 mg; excipient wheat middlings

^9^Ascorbil monophosphate, PREMIX Lda, Portugal

^10^Monosodium phosphate, Fosfitalia, Italy

^11^l-Taurine 98.5%: Ajinomoto Eurolysine SAS, France

^12^Mixture of glutamic acid 8.5 mg 100 g^−1^, l-histidine HCl H_2_O 232.8 mg 100 g^−1^, and inosine-5/-monophosphate 2Na 200.9 mg 100 g^−1^; Premix Lda, Castelo de Neiva, Portugal

^13^Proprietary product, Sparos Lda., Olhão, Portugal

^14^Premix Lda, Castelo de Neiva, Portugal

^15^Proprietary binder mix, Sparos Lda., Olhão, Portugal

**Table 2:** Analyzed proximate composition (% of dry mass), gross energy (kJ g^−1^), α-tocopherol, ascorbic acid (mg kg^−1^), taurine (mg g^−1^), and mineral (macroelements as mg g^−1^ and microelements as μg g^−1^) contents of the reference and test diets

	MGK	15KO	20KO	15KORO
Crude protein (% DM)	55.7	56.3	57.9	56.4
Crude lipid (% DM)	17.9	14.3	18.9	14.7
Protein/lipid	3.1	3.9	3.1	3.8
Carbohydrate (% DM)	14.9	11.7	9.1	7.0
Ash (% DM)	7.2	10.4	10.7	10.1
Gross energy (kJ g^−1^)	21.8	20.2	21.3	20.2
α-Tocopherol (mg kg^−1^)	1479.6	311.6	341.0	252.7
Ascorbic acid (mg kg^−1^)	1852.7	908.2	918.6	868.1
Taurine (mg g^−1^)	6.1	28.1	23.8	26.4
Macrominerals (mg g^−1^)				
Sodium	7.0	1.3	1.3	1.3
Magnesium	1.6	3.0	3.4	3.3
Phosphorus	12.8	18.7	19.7	9.6
Potassium	10.4	6.5	6.6	6.6
Calcium	9.5	9.8	10.1	10.1
Microminerals (μg g^−1^)				
Chromium	1.0	18.8	29.9	24.9
Manganese	66.2	25.8	29.7	41.1
Iron	582.5	578.3	762.9	601.2
Cobalt	0.7	0.9	1.2	1.2
Copper	11.1	24.3	27.6	30.7
Zinc	208.4	60.4	90.0	93.6
Selenium	6.3	12.3	12.0	12.0

Results are means of duplicate analyses

*MGK* reference diet (Magokoro Nishin Marubeni®, Japan), *15KO* 15% lipid as krill oil, *20KO* 20% lipid as krill oil, *15KORO* 15% lipid (1:1 krill oil and rapeseed oil)

Total lipid fatty acid composition, expressed as percent total fatty acid or micrograms per milligram dry mass, of the reference and experimental diets are presented in Table 3. Diets based on krill oil alone (15KO and 20KO) showed the highest values for total n−3 PUFA (41.5% and 43.2%, respectively), with levels of 22:6n−3 (DHA) 22% and 22.6%, respectively, and 20:5n−3 (EPA) 13.3% and 14.2%, respectively. Lowest values for these fatty acids were found in diet 15KORO, although similar to those in the reference MGK diet. In absolute terms, the content of DHA ranged from 15.5 μg mg^−1^ diet dry mass in diet 15KORO up to 29.5 μg mg^−1^ diet dry mass in diet 20KO with the reference and 15KO diets showing intermediate levels of 20.4 and 22.6 μg of DHA per milligram diet dry mass, respectively. Total saturated fatty acids, primarily 16:0, were about 28% of total fatty acids in all diets except diet 15KORO that showed only 20% while, in contrast, monoenes were highest in this diet (~ 40% due to the high content of 18:1n−9). The inclusion of 50% RO in diet 15KORO resulted in this diet presenting the highest values for 18:2n−6 (LA) (7.8%) and 18:3n−3 (LNA) (3.9%).

**Table 3:** Total lipid fatty acid composition, expressed as % total fatty acid and μg mg^−1^ mass (in brackets), of the reference and experimental diets

	MGK	15KO	20KO	15KORO
14:0	5.3 (6.6)	6.8 (6.9)	7.0 (9.2)	3.6 (3.6)
16:0	17.8 (22.3)	16.5 (16.9)	16.5 (21.5)	12.1 (12.1)
18:0	4.7 (5.9)	4.3 (4.4)	3.4 (4.4)	4.2 (4.1)
Total SFA^1^	29.1 (36.3)	28.3 (28.9)	27.5 (35.8)	20.6 (20.6)
16:1n−7	4.9 (6.2)	4.9 (5.0)	5.0 (6.5)	3.0 (3.0)
18:1n−9	13.5 (16.9)	10.9 (11.1)	10.3 (11.5)	27.0 (26.9)
18:1n−7	3.3 (4.1)	4.6 (4.7)	4.9 (6.3)	3.9 (3.9)
20:1n−9	1.9 (2.4)	2.2 (2.3)	1.9 (2.4)	2.5 (2.5)
Total MUFA^2^	30.7 (38.4)	26.0 (26.6)	25.2 (32.9)	39.7 (39.5)
18:2n−6	5.6 (7.0)	2.2 (2.2)	2.0 (2.6)	7.8 (7.8)
20:4n−6	1.1 (1.4)	0.6 (0.6)	0.6 (0.7)	0.5 (0.5)
Total n−6 PUFA^3^	7.7 (9.6)	3.4 (3.4)	3.1 (4.1)	8.7 (8.7)
18:3n−3	1.2 (1.5)	0.9 (1.0)	0.9 (1.2)	3.9 (3.9)
18:4n−3	1.8 (2.2)	2.0 (2.0)	2.1 (2.7)	1.1 (1.1)
20:4n−3	0.7 (0.9)	0.4 (0.4)	0.4 (0.5)	0.3 (0.3)
20:5n−3	8.9 (11.1)	13.3 (13.6)	14.2 (18.5)	7.7 (7.7)
22:5n−3	2.4 (3.0)	2.7 (2.8)	2.8 (3.7)	2.0 (2.0)
22:6n−3	16.3 (20.4)	22.0 (22.6)	22.6 (29.5)	15.5 (15.5)
Total n−3 PUFA^4^	31.5 (39.3)	41.5 (42.5)	43.2 (56.4)	30.5 (30.5)
Total PUFA	40.2 (50.2)	45.8 (46.9)	47.3 (61.7)	39.7 (39.7)
n−3/n−6	4.1	12.2	13.9	3.5
DHA/EPA	1.8	1.6	1.6	2.0

Results are means of duplicate analyses. ^1^Totals include 15:0, 20:0, 22:0, and 24:0. ^2^Totals include 16:1n−9, 18:1n−11, 20:1n−7, 22:1 isomers, and 24:1. ^3^Totals include 18:3n−6, 20:2n−6, 22:4n−6, and 22:5n−6. ^4^Totals include 20:3n−3 and 22:3n−3

*DHA* docosahexaenoic acid, *EPA* eicosapentaenoic acid, *MGK* reference diet (Magokoro Nishin Marubeni®, Japan), *MUFA* monounsaturated fatty acid, *PUFA*, polyunsaturated fatty acid, *SFA* saturated fatty acid, *15KO* 15% total lipid as krill oil, *20KO* 20% total lipid as krill oil, *15KORO* 15% total lipid (1:1 krill oil and rapeseed oil)

### Experimental protocol

Prior to initiation of the feeding trials, from 32 to 41 dah, ABT juveniles were fed with a 1:1:1 mixture of MGK, 15KO, and 20KO for trial A, and MGK, 15KO, and 15KORO for trial B. Both trials were carried out in duplicate tanks per treatment, with each tank containing either 40 or 46 ABT juveniles in trials A and B, respectively, with fish fed one of the dietary treatments, pellet size 1.2 mm, from 41 to 51 dah when trials were terminated and fish sampled. Thus, six cylindroconical 5-m^3^ tanks were used in an open flow system with incoming seawater filtered at 10 μm and UV sterilized. Rearing conditions during the experimental period are shown in Supplementary Table 1. Fish were hand-fed to satiation every 2 h, during the light hours of the photoperiod, eight times per day. The feeding trials and all experimental (sampling) procedures were carried out according to Spanish (RD 53/2013; BOE 8th February 2013) and EU legislation (Directive 2010/63/EU) on the protection of animals used for scientific purposes. The trials were also subject to ethical review by the Animal Welfare and Ethical Review Board of the University of Stirling. In this respect, it is important to note that the trials were run for the minimum duration (10 days) required to produce a 2- to 3-fold increase in weight, which is the generally accepted standard for fish nutritional trials. This was because the major cause of death in ABT juveniles in tanks at this stage is collision with the tank walls (Miyashita et al. 2000), and so the trial duration was limited by considerations of fish welfare. However, similarly short trials testing dietary oil sources have been successfully performed with juvenile PBT (Agawa et al. 2012).

### Sampling for biometrical, biochemical, and molecular analysis

At the initiation of the feeding trials (41 dah), 30 randomly caught ABT juveniles were euthanized by a lethal dose of anesthetic (0.02% 2-phenoxyethanol; Sigma, Spain), weights and total lengths recorded, and individual fish photographed while measuring. At the end of the trials (51 dah), surviving ABT juveniles from all treatment replicates were measured and weighed with final survival (%) calculated by counting juveniles at the beginning and end of the trial from every replicate tank. Growth performance and feed utilization response variables including mean weight (g), mean length (cm), percent weight gain (%), specific growth rate (SGR) as percentage of daily growth increase, feed conversion ratio (FCR), condition factor (CF), and daily feeding rate (DFR, %) were calculated using the following formulae.

$$ {\displaystyle \begin{array}{l}\begin{array}{l}\mathrm{Weight}\ \mathrm{gain}\ \left(\%\right)=\left(\mathrm{average}\ \mathrm{weight}\ \mathrm{gain}/\mathrm{average}\ \mathrm{initial}\ \mathrm{body}\kern0.17em \mathrm{weight}\right)\times 100,\mathrm{where}\ \mathrm{average}\ \mathrm{SGR}\left(\%{\mathrm{day}}^{-1}\right)=\\ {}\left[\left(\ln {W}_2-\ln {W}_1\right)/\mathrm{time}\left(\mathrm{days}\right)\right]\times 100,\mathrm{where}\ {W}_1\ \mathrm{and}\ {W}_2\ \mathrm{denote}\ \mathrm{the}\ \mathrm{initial}\ \mathrm{and}\ \mathrm{final}\ \mathrm{weight}\left(\mathrm{g}\right),\mathrm{respectively}.\end{array}\\ {}\mathrm{FCR}=\mathrm{dry}\ \mathrm{feed}\ \mathrm{intake}\ \left(\mathrm{g}\right)/\mathrm{wet}\ \mathrm{weight}\ \mathrm{gain}\left(\mathrm{g}\right).\\ {}\mathrm{CF}=\left(W/{L}^3\right)\times 100,\mathrm{where}\ W\;\mathrm{and}\;L\;\mathrm{denote}\ \mathrm{wet}\ \mathrm{body}\ \mathrm{weight}\left(\mathrm{g}\right)\mathrm{and}\ \mathrm{fork}\ \mathrm{length}\left(\mathrm{cm}\right),\mathrm{respectively}.\\ {}\mathrm{DFR}\ \left(\%\right)=\mathrm{feed}\ \mathrm{intake}\left(\mathrm{dry}\ \mathrm{matter}\right)/100/\left[\left(\mathrm{initial}\ \mathrm{fish}\ \mathrm{weight}+\mathrm{final}\ \mathrm{fish}\ \mathrm{weight}\right)10\ \mathrm{days}\ \mathrm{fed}/2\right].\end{array}} $$

At the end of the feeding trials and after total weight and length were measured, livers and/or small pieces of muscular tissue of three individual ABT juveniles per tank were dissected and preserved for biochemical and molecular analyses. For molecular analyses, approximately 100–150 mg of liver tissue (samples of individual livers from three fish per tank; six per dietary treatment) were placed in 1 ml RNA*later*® (Sigma-Aldrich, Dorset, UK) and processed according to the manufacturer’s instructions (4 °C for 24 h) before storage at − 80 °C for RNA extraction and subsequent molecular analysis. Duplicate samples of livers and muscle per tank (pooled samples of liver/muscle from three fish per tank, two per dietary treatment) were immediately frozen in liquid N_2_ and stored at − 80 °C prior to biochemical analysis.

### Biochemical analysis

#### Proximate composition of diets

Gross proximate compositions of feeds (protein, lipid, ash, and moisture) were determined according to standard procedures (AOAC 2000). Moisture contents were obtained after drying in an oven at 110 °C for 24 h and ash content determined after incineration at 600 °C for 16 h. Crude protein was measured by determining nitrogen content (N × 6.25) using automated Kjeldahl analysis (Tecator Kjeltec Auto 1030 analyzer; Foss, Warrington, UK) and crude lipid content determined gravimetrically after Soxhlet lipid extraction (Tecator Soxtec system 2050 Auto Extraction apparatus).

#### Total lipid extraction and quantification and fatty acid analysis

Total lipid was extracted from feeds, pooled livers, and muscular tissue (livers/muscle of three fish per tank pooled) of ABT juveniles fed the different dietary regimes according to the method of Folch et al. (1957). Approximately 200 mg of ground feed or hepatic tissue was placed in ice-cold chloroform/methanol (2:1, by vol) and homogenized with an Ultra-Turrax tissue disrupter (Fisher Scientific, Loughborough, UK). The non-lipid and lipid layers were separated by addition of 0.88% (*w*/*v*) KCl and allowed to separate on ice for 1 h. The upper aqueous layer was aspirated and the lower organic layer dried under oxygen-free nitrogen. The lipid content was determined gravimetrically after drying overnight in a vacuum desiccator.

Fatty acid methyl esters (FAMEs) of total lipid were prepared by acid-catalyzed trans-esterification at 50 °C for 16 h according to the method of Christie (2003). The FAMEs were separated and quantified by gas–liquid chromatography (Agilent Technologies 7890B GC System) using a 30 m × 0.32 mm i.d. fused silica capillary column (SUPELCOWAX™-10; Supelco Inc., Bellefonte, USA) and on-column injection at 50 °C. Hydrogen was used as carrier gas and temperature programming was from 50 to 150 °C at 40 °C/min and then to 230 °C at 2.0 °C/min. Individual methyl esters were identified by comparison with known standards and by reference to published data (Ackman 1980; Tocher and Harvie 1988). Data were collected and processed using Agilent Technologies Openlab CDS Chemstation for Windows (version A.02.05.21).

#### Determination of alpha-tocopherol (vitamin E), ascorbic acid (vitamin C), and taurine contents in reference and test diets

Alpha-tocopherol concentrations in reference and test diets were determined using high-pressure liquid chromatography (HPLC) with UV detection. Samples were weighed, homogenized in pyrogallol, and saponified essentially as described by McMurray et al. (1980) according to Cowey et al. (1981). HPLC analysis was performed using a 150 × 4.6 mm, reverse-phase Luna 5 μm C18 column (Phenomenex, CA, USA). The mobile phase was 98% methanol pumped at 1.0 ml min^−1^. The effluent from the column was monitored at a wavelength of 293 nm and quantification achieved by comparison with alpha-tocopherol (Sigma-Aldrich) as external standard.

The concentration of vitamin C was determined as described by Betancor et al. (2012a). Samples were weighed, homogenized, and dissolved in 0.4 M phosphate buffer (adjusted to pH 3.0 with phosphoric acid). The samples were centrifuged at 1500x*g*, supernatants removed and filtered through a disposable 0.45-μm filter and stored at 4 °C until the measurement in a HPLC with UV detection. The determination of vitamin C concentration was achieved by comparison with tris (cyclohexylammonium) ascorbic acid-2-phosphate (Sigma-Aldrich) as the external standard.

Taurine in feeds was analyzed by reverse-phase HPLC using a Nova Pak C18 column (3.9 × 300 mm) (Waters Corporation, MA, USA) fitted with a Nova Pack C18 precolumn (3.9 × 20 mm) according to the method of Bidlingmeyer et al. (1987). Separation was achieved over 65 min at 52 **°**C with a flow rate of 1 ml min^−1^, using a gradient between two solvents: 70 mM sodium acetate at pH 6.55 with 2.5% of acetonitrile (solvent A) and water–acetonitrile–methanol, 40:45:15 *v*/*v* (solvent B) with UV detection at 254 nm.

#### Mineral analysis

Total selenium and other minerals in feeds were measured according to the methods described in Betancor et al. (2012b) using inductively coupled plasma mass spectrometry (ICP-MS) with collision cell technology (Thermo X Series 2; Thermo Scientific, Hemel-Hempstead, UK) using argon and hydrogen as carrier gases. Briefly, between 50 and 100 mg of ground feed sample was added to Teflon tubes and digested in a microwave digester (MARS Xpress; CEM Microwave Technology Ltd., Buckingham, UK) with 5 ml of 69% nitric acid in three stages: 21–190 °C for 10 min at 800 W followed by 190 °C for 20 min at 800 W followed by a final 30-min cooling period. The digested solution was made up to a 10-ml volume with deionized water and total minerals determined by adding 0.4 ml of this solution to 10-ml tubes and adjusting volume (10 ml) using deionized water before analyzing by ICP-MS. For total selenium, 10 μl of internal standard (aqueous solution of gallium and scandium, 10 ppm; BDH Chemicals Ltd., Poole, UK) and 0.2 ml methanol were added to 0.4 ml of the initial digest before adjusting the volume to 10 ml with deionized water prior to analysis by ICP-MS.

### Tissue RNA extraction and cDNA synthesis

Samples were homogenized in 1 ml of TriReagent® (Sigma-Aldrich) RNA extraction buffer using a bead tissue disruptor (Bio Spec, Bartlesville, Oklahoma, USA). Total RNA was isolated following manufacturer’s instructions and quantity and quality determined by spectrophotometry using a Nanodrop ND-1000 (Labtech Int., East Sussex, UK), and electrophoresis using 200 ng of total RNA in a 1% agarose gel. cDNA was synthesized using 2 μg of total RNA and random primers in 20-μl reactions and the high-capacity reverse transcription kit without RNase inhibitor according to the manufacturer’s protocol (Applied Biosystems, Warrington, UK).

### Quantitative RT-polymerase chain reaction analysis

Transcript abundance was determined by quantitative RT-polymerase chain reaction (qPCR) of candidate genes involved in key pathways related to lipid and fatty acid metabolism, and antioxidant system enzymes. Specifically, qPCR was carried out on transcription factors *pparα*, *pparγ*, *lxr*, *rxr*, *srebp1*, and *srebp2*; LC-PUFA biosynthesis genes *fads2d6* and *elovl5*; fatty acid metabolism genes *fas*, *cpt1*, *aco*, *fabp2*, *fabp4*, *fabp7*, *lpl*, and *hmgcl*; and the antioxidant enzymes *sod, cat, gpx1*, and *gpx4*. *Elongation factor-1α* (*elf1α*) and *β-actin* (*bactin*) were used as reference genes. The cDNA was diluted 20-fold with Milli-Q water. The efficiency of the primers for each gene was previously evaluated by serial dilutions of cDNA pooled from the samples to guarantee it was > 85% for all primer pairs. qPCR was performed using a Biometra TOptical Thermocycler (Analytik Jena, Goettingen, Germany) in 96-well plates in duplicate 20 μl reaction volumes containing 10 μl of Luminaris Color HiGreen qPCR Master Mix (Thermo Scientific, Hemel Hempstead, UK), 1 μl of the primer corresponding to the analyzed gene (10 pmol), 3 μl of molecular biology grade water, and 5 μl of cDNA (1/20 diluted). In addition, amplifications were carried out with a systematic negative control (NTC, no template control) containing no cDNA. Standard amplification parameters included an UDG pre-treatment at 50 °C for 2 min, an initial denaturation step at 95 °C for 10 min, followed by 35 cycles: 15 s at 95 °C, 30 s at the annealing *T*_m_, and 30 s at 72 °C. Primer sequences for genes are given in Table S2 (Supplementary material).

### Statistical analysis

Results for biometry, growth, and feed utilization response variables and fatty acid compositions are presented as means ± SD with *n* = 2 for biochemical analyses or, with the accepted compromise of pseudoreplication, *n* = 6 for gene expression. The data were checked for homogeneity of the variances by the Bartlett test and, where necessary, arc-sin transformed before further statistical analysis. Differences between mean values were analyzed by *t* test and one-way analysis of variance (ANOVA) followed, when pertinent, by a multiple comparison test (Tukey). Differences rejecting the null hypothesis were reported as statistically significant when *P* < 0.05 (Zar 1999). Gene expression results were analyzed using the relative expression software tool (REST 2009), which employs a pairwise fixed reallocation randomization test (10,000 randomizations) with efficiency correction (Pfaffl et al. 2002) to determine the statistical significance of expression ratios (gene expression fold changes) among treatments.

## Results

### ABT juvenile biometry, growth performance, feed utilization response, and survival

Growth performance, feed utilization, and survival of ABT juveniles fed the reference and experimental diets are shown in Table 4. In trial A, the greatest total length was shown for ABT juveniles fed MGK and 15KO diets with the lowest value for fish fed 20KO. Fish fed MGK also displayed the highest total wet weight, total weight gain, and SGR followed by fish fed 15KO and those fed 20KO. Fish fed 15KO and MGK showed higher condition factor (CF). In contrast, feed utilization parameters such as feed conversion ratio (FCR), daily feeding rate (DFR), ingestion rate (IR), and survival did not show significant differences among treatments. In trial B, total wet weight and length were higher in fish fed diets 15KO and 15KORO and lower in those fed the MGK diet. The same pattern was shown for weight gain and SGR, while CF was higher in fish fed MGK and 15KO, and lower in those fed the 15KORO diet. As in trial A, in trial B feed utilization parameters and survival did not present significant differences among fish fed any of the diets (Table 4). Mortality was relatively high, as expected for ABT of this size in cylindroconical 5-m^3^ tanks, and almost exclusively due to collisions with the tank wall and not related to feed (Miyashita et al. 2000).

**Table 4:** Growth performance, feed utilization, and survival of juvenile Atlantic bluefin tuna (*Thunnus thynnus*) fed reference and test diets

Trial A			
Dietary treatments			
Parameter measured	MGK	15KO	20KO
Initial wet weight (g)	2.9 ± 0.9	2.9 ± 0.9	2.9 ± 0.9
Final wet weight (g)	8.4 ± 0.3^a^	8.0 ± 0.2^b^	6.0 ± 0.3^c^
Initial fork length (cm)	6.5 ± 0.7	6.5 ± 0.7	6.5 ± 0.7
Final fork length (cm)	8.5 ± 0.1^a^	8.5 ± 0.1^a^	8.1 ± 0.1^b^
Weight gain (%)	188.4 ± 16.3^a^	177.1 ± 13.6^b^	107.5 ± 8.8^c^
Specific growth rate (SGR %·day^−1^)	19.4 ± 0.4^a^	19.0 ± 0.2^b^	15.5 ± 0.5^c^
Condition factor (CF g/cm^3^)	1.3 ± 0.1^a^	1.3 ± 0.1^a^	1.1 ± 0.1^b^
Feed conversion ratio (FCR)	0.8 ± 0.0	1.0 ± 0.0	0.9 ± 0.1
Daily feeding rate (DFR %)	6.5 ± 0.5	7.3 ± 0.7	8.4 ± 0.5
Survival (%)	25.0 ± 10.6	35.6 ± 11.5	15.0 ± 0.1
Trial B			
Dietary treatments			
Parameter measured	MGK	15KO	15KORO
Initial wet weight (g)	3.3 ± 0.6	3.3 ± 0.6	3.3 ± 0.6
Final wet weight (g)	10.4 ± 0.3^b^	11.3 ± 0.1^a^	11.2 ± 0.1^a^
Initial fork length (cm)	6.7 ± 0.4	6.7 ± 0.4	6.7 ± 0.4
Final fork length (cm)	9.1 ± 0.1^b^	9.4 ± 0.1^a^	9.4 ± 0.1^a^
Weight gain (%)	216.1 ± 16.4^b^	243.5 ± 8.2^a^	242.2 ± 19.6^a^
Specific growth rate (SGR %·day^−1^)	22.1 ± 0.2^b^	22.9 ± 0.1^a^	23.0 ± 0.2^a^
Condition factor (CF g/cm^3^)	1.4 ± 0.1^a^	1.4 ± 0.1^a^	1.3 ± 0.1^b^
Feed conversion ratio (FCR)	1.0 ± 0.0	1.0 ± 0.0	1.1 ± 0.2
Daily feeding rate (DFR %)	6.0 ± 0.5	6.3 ± 0.3	6.7 ± 0.3
Survival (%)	23.8 ± 6.1	26.8 ± 1.0	25.0 ± 4.6

Results are mean ± SD (*n* = 2, with 20 animals measured per replicate). An SD of 0.0 implies an SD of < 0.05. Values bearing different superscript letters are significantly different (*P* < 0.05)

*MGK* reference diet (Magokoro®, Marubeni Nisshin Feed Co., Japan), *15KO* diet containing 15% total lipid as krill oil, *20KO* diet containing 20% total lipid as krill oil, *15KORO* diet containing 15% total lipid as 50% krill oil and 50% rapeseed oil

### Lipid contents and fatty acid compositions of total lipid of liver and muscle from ABT juveniles

In trial A, lipid content (% of wet mass) of liver was highest in fish fed MGK (6.5%), followed by those fed the 15KO diet (3.4%) and lowest in liver of fish fed the 20KO diet (2.2%) (Table 5). The proportion of total n−3 PUFA was highest in livers of fish fed the 15KO and 20KO diets, mainly due to high proportions of DHA with the highest in liver from fish fed 20KO followed by 15KO, and lowest in fish fed diet MGK. Total n−6 PUFA content was highest in livers of fish fed MGK, mainly due to the high levels of 18:2n−6 (LA) in this diet, followed by livers of fish fed the 20KO diet, which also showed the highest level of 20:4n−6 (ARA), followed by livers of fish fed 15KO. The percentage of total saturated fatty acids, primarily 16:0, in liver total lipid was highest in fish fed diet 20KO followed by fish fed 15KO and MGK, whereas total monoenes were highest (30%) in livers of fish fed the MGK diet, primarily due to levels of 18:1n−9 (15.7%), with levels in 15KO > 20KO.

**Table 5:** Lipid contents (% of wet weight) and fatty acid compositions (% of total fatty acids) of liver and muscle from juvenile Atlantic bluefin tuna (*Thunnus thynnus* L.) fed either a reference diet (Magokoro®), 15% lipid diet based on krill oil (15KO), or 20% lipid diet based on krill oil (20KO) in trial A

	Liver			Muscle		
	MGK	15KO	20KO	MGK	15KO	20KO
Total lipid (%)	6.5 ± 0.8^a^	3.4 ± 0.7^b^	2.2 ± 0.1^c^	0.8 ± 0.2	0.8 ± 0.1	0.8 ± 0.0
14:0	2.5 ± 0.1^a^	1.6 ± 0.3^b^	0.5 ± 0.1^c^	0.8 ± 0.1^a^	0.7 ± 0.1^a^	0.3 ± 0.0^b^
16:0	18.0 ± 0.7^c^	20.6 ± 0.2^b^	24.7 ± 1.0^a^	19.6 ± 0.2^a^	20.5 ± 1.9^a^	17.3 ± 0.8^b^
18:0	6.3 ± 0.2^b^	6.8 ± 0.1^b^	11.4 ± 0.5^a^	10.9 ± 0.3^b^	10.5 ± 0.4^b^	14.1 ± 0.4^a^
Total SFA^1^	28.2 ± 1.1^b^	30.1 ± 0.2^b^	38.8 ± 1.0^a^	33.1 ± 1.1	33.6 ± 1.9	34.3 ± 0.7
16:1n−7	3.7 ± 0.2^a^	2.6 ± 0.2^b^	1.1 ± 0.1^c^	1.3 ± 0.2^a^	1.2 ± 0.2^a^	0.7 ± 0.1^b^
18:1n−9	15.6 ± 0.7^a^	9.6 ± 0.3^b^	5.8 ± 1.3^c^	9.0 ± 0.4^a^	8.0 ± 0.1^b^	7.8 ± 0.6^b^
18:1n−7	4.1 ± 0.2^a^	4.5 ± 0.1^a^	3.0 ± 0.3^b^	3.0 ± 0.2^b^	3.8 ± 0.1^a^	3.7 ± 0.1^a^
20:1n−9	2.0 ± 0.1^a^	1.2 ± 0.1^b^	0.3 ± 0.0^c^	0.7 ± 0.1^a^	0.9 ± 0.1^a^	0.5 ± 0.0^b^
Total MUFA^2^	30.0 ± 1.2^a^	19.8 ± 0.3^b^	12.1 ± 2.2^c^	17.8 ± 0.4^a^	15.0 ± 0.1^b^	13.7 ± 0.4^b^
18:2n−6	6.9 ± 0.3^a^	1.8 ± 0.2^b^	0.9 ± 0.1^c^	3.3 ± 0.1^a^	1.4 ± 0.1^b^	1.3 ± 0.1^b^
20:4n−6	1.7 ± 0.1^b^	1.3 ± 0.1^c^	2.4 ± 0.1^a^	2.0 ± 0.1^a^	1.2 ± 0.1^c^	1.6 ± 0.1^b^
22:5n−6	0.7 ± 0.1	0.9 ± 0.1	1.1 ± 0.1	1.6 ± 0.1^a^	1.0 ± 0.1^b^	1.2 ± 0.2^b^
Total n−6 PUFA^3^	11.3 ± 0.4^a^	5.7 ± 0.3^c^	6.8 ± 0.6^b^	8.2 ± 0.1^a^	5.6 ± 0.3^c^	6.6 ± 0.1^b^
18:3n-3	0.8 ± 0.1^a^	0.5 ± 0.0^b^	0.2 ± 0.0^c^	0.3 ± 0.0^a^	0.3 ± 0.0^a^	0.2 ± 0.0^b^
18:4n−3	0.9 ± 0.1^a^	0.6 ± 0.2^a^	0.3 ± 0.0^b^	0.3 ± 0.0^a^	0.3 ± 0.0^a^	0.2 ± 0.0^b^
20:4n−3	0.5 ± 0.0^a^	0.3 ± 0.0^b^	0.2 ± 0.0^b^	0.3 ± 0.0^a^	0.2 ± 0.0^b^	0.2 ± 0.0^b^
20:5n−3	6.1 ± 0.4^b^	7.8 ± 0.7^a^	5.7 ± 0.4^b^	5.3 ± 0.2^c^	7.6 ± 0.3^a^	6.5 ± 0.2^b^
22:5n−3	2.1 ± 0.2^a^	2.1 ± 0.2^a^	0.8 ± 0.0^b^	1.6 ± 0.1^a^	1.5 ± 0.2^ab^	1.2 ± 0.0^b^
22:6n−3	15.1 ± 1.3^b^	27.0 ± 0.8^a^	28.8 ± 0.9^a^	28.3 ± 0.3^b^	31.9 ± 0.8^a^	31.2 ± 0.5^a^
Total n−3 PUFA^4^	27.1 ± 2.0^b^	39.6 ± 0.2^a^	37.5 ± 1.3^a^	37.6 ± 0.2^b^	42.7 ± 2.2^a^	40.7 ± 1.8^a^
C16 PUFA	2.4 ± 0.1^a^	1.7 ± 0.0^b^	2.6 ± 0.1^a^	2.0 ± 0.1^a^	2.0 ± 0.0^b^	2.4 ± 0.1^a^
Total PUFA	38.4 ± 2.3^b^	45.4 ± 0.4^a^	44.4 ± 1.8^a^	45.8 ± 1.3	48.3 ± 2.4	47.3 ± 1.9
DHA/EPA	2.5 ± 0.1^c^	3.5 ± 0.4^b^	5.1 ± 0.2^a^	5.3 ± 0.1^a^	4.2 ± 0.1^c^	4.8 ± 0.1^b^
n−3/n−6	2.4 ± 0.1^c^	6.9 ± 0.3^a^	5.5 ± 0.3^b^	4.6 ± 0.2^c^	7.6 ± 0.2^a^	6.2 ± 0.3^b^
Unknown	3.4 ± 0.3^b^	4.7 ± 0.2^a^	4.7 ± 0.4^a^	3.3 ± 0.2^ab^	3.1 ± 0.2^b^	4.7 ± 0.3^a^

Results are means ± SD (*n* = 2). An SD of 0.0 implies an SD of < 0.05. Mean values, within the same dietary trial, bearing different superscript letters are significantly different (*P* < 0.05). ^1^Totals include 15:0, 20:0, 22:0, and 24:0. ^2^Totals include 16:1n−9, 18:1n−11, 20:1n−7, 22:1 isomers, and 24:1. ^3^Totals include 18:3n−6, 20:2n−6, and 22:4n−6. ^4^Totals include 20:3n−3 and 22:3n−3

*DHA* docosahexaenoic acid, *EPA* eicosapentaenoic acid, *MGK* Magokoro, *MUFA* monounsaturated fatty acid, *PUFA* polyunsaturated fatty acid, *SFA* saturated fatty acid, *15KO* diet containing 15% lipid as krill oil, *20KO* diet containing 20% lipid as krill oil, *15KORO* diet containing 15% lipid as 50% krill oil and 50% rapeseed oil

In trial B, lipid content of liver was highest in fish fed the reference MGK diet (10.0%) followed by those fed 15KORO and 15KO diets at 3.7% and 5.4%, respectively (Table 6). Total saturated fatty acids were highest in liver from fish fed the MGK and 15KO diets (27.2% and 29.4%, respectively), primarily due to the levels of 16:0, and lowest in those of fish fed the 15KORO diet (Table 6). Total monoenes in liver total lipid were highest in fish fed the MGK and 15KORO diets (30.8% and 33.4%, respectively), due to the levels of 18:1n−9, which was highest in liver of fish fed diet 15KORO (23.7%), followed by liver of fish fed the MGK diet (16.1%) and lowest in fish fed diet 15KO (10.5%). Liver of fish fed the MGK and 15KORO diets showed the highest values of total n−6 PUFA (11.6% and 11.0%, respectively) mainly due to high levels of LA (7.4% and 7.5%, respectively), reflecting the inclusion of rapeseed oil, with lowest level in fish fed the 15KO diet. Highest ARA levels were observed in total lipid of livers from fish fed the reference MGK diet, followed by those fed the 15KORO and 15KO diets. The proportion of total n−3 PUFA was highest in liver of fish fed the 15KO diet (37.6%) followed by those fed MGK and 15KORO diets at 26.9% and 25.5%, respectively. This was mainly due to the contribution of DHA with proportions of 23.5%, 15.4%, and 13.7% for fish fed the 15KO, 15KORO, and MGK diets, respectively, reflecting to a great extent dietary fatty acid compositions. The DHA/EPA ratio in liver lipid was, in decreasing order, 2.9 (15KORO), 2.6 (15KO), and 2.1 (MGK).

**Table 6:** Lipid contents (% of wet weight) and fatty acid compositions (% of total fatty acids) of liver and muscle from juvenile Atlantic bluefin tuna (*Thunnus thynnus* L.) fed either a reference diet (Magokoro®), 15% lipid diet based on krill oil (15KO), or 15% total lipid diet based on krill oil and rapeseed oil 1:1 (15KORO) in trial B

	Liver			Muscle		
	MGK	15KO	15KORO	MGK	15KO	15KORO
Total lipid (%)	10.0 ± 1.0^a^	3.7 ± 2.2^b^	5.4 ± 1.9^b^	0.9 ± 0.0	0.9 ± 0.0	0.9 ± 0.3
14:0	3.3 ± 0.1^a^	2.4 ± 0.4^b^	1.5 ± 0.4^c^	1.2 ± 0.4^a^	1.4 ± 0.3^a^	0.8 ± 0.1^b^
16:0	17.2 ± 0.6^ab^	19.7 ± 0.7^a^	14.8 ± 2.3^b^	18.8 ± 1.1	20.8 ± 0.3	18.1 ± 0.3
18:0	5.5 ± 0.4	5.6 ± 0.9	5.6 ± 0.7	9.7 ± 0.8^a^	9.4 ± 0.2^ab^	8.8 ± 0.1^b^
Total SFA^1^	27.2 ± 1.0^ab^	29.4 ± 0.8^a^	23.3 ± 2.5^b^	31.5 ± 1.3^ab^	33.4 ± 0.9^a^	29.5 ± 0.5^b^
16:1n−7	4.3 ± 0.2^a^	3.4 ± 0.5^ab^	2.0 ± 0.5^b^	1.6 ± 0.3^a^	1.6 ± 0.1^a^	0.9 ± 0.1^b^
18:1n−9	16.1 ± 0.9^b^	10.5 ± 1.1^c^	23.7 ± 4.3^a^	9.8 ± 0.2^b^	7.8 ± 0.5^c^	14.4 ± 1.0^a^
18:1n−7	4.0 ± 0.2^b^	5.2 ± 0.6^a^	4.3 ± 0.2^b^	2.9 ± 0.1^c^	3.8 ± 0.3^a^	3.2 ± 0.0^b^
20:1n−9	1.9 ± 0.1^a^	1.2 ± 0.1^b^	1.4 ± 0.3^ab^	0.9 ± 0.0	0.8 ± 0.0	1.1 ± 0.1
Total MUFA^2^	30.8 ± 1.6^a^	22.2 ± 2.4^b^	33.4 ± 5.3^a^	18.3 ± 0.4^a^	15.1 ± 0.2^b^	20.8 ± 1.4^a^
18:2n−6	7.4 ± 0.4^a^	2.2 ± 0.3^b^	7.5 ± 1.3^a^	3.9 ± 0.1^b^	1.6 ± 0.1^c^	4.5 ± 0.2^a^
20:4n−6	1.5 ± 0.1^a^	1.0 ± 0.2^b^	1.1 ± 0.3^ab^	1.9 ± 0.2^a^	1.1 ± 0.0^b^	1.2 ± 0.1^b^
22:5n−6	0.7 ± 0.1^a^	0.9 ± 0.1^a^	0.4 ± 0.2^b^	1.1 ± 0.1^a^	0.6 ± 0.1^b^	0.6 ± 0.0^b^
Total n−6 PUFA^3^	11.6 ± 0.6^a^	6.3 ± 0.2^b^	11.0 ± 1.2^a^	9.3 ± 0.5^a^	5.9 ± 0.2^b^	8.6 ± 0.1^a^
18:3n−3	1.0 ± 0.0^b^	0.6 ± 0.1^c^	1.9 ± 0.5^a^	0.4 ± 0.0^b^	0.3 ± 0.0^b^	1.0 ± 0.2^a^
18:4n−3	1.1 ± 0.0^a^	1.0 ± 0.1^a^	0.5 ± 0.2^b^	0.4 ± 0.1^a^	0.4 ± 0.0^a^	0.2 ± 0.0^b^
20:4n−3	0.6 ± 0.0^a^	0.3 ± 0.0^b^	0.2 ± 0.1^b^	0.3 ± 0.0^a^	0.2 ± 0.0^b^	0.2 ± 0.0^b^
20:5n−3	6.6 ± 0.3^b^	9.0 ± 0.3^a^	5.3 ± 0.2^c^	5.9 ± 0.1^b^	8.1 ± 0.2^a^	6.1 ± 0.2^b^
22:5n−3	2.4 ± 0.2^a^	2.0 ± 0.2^a^	1.4 ± 0.3^b^	1.8 ± 0.0^a^	1.5 ± 0.1^b^	1.5 ± 0.0^b^
22:6n−3	13.7 ± 0.6^b^	23.5 ± 2.3^a^	15.4 ± 4.2^b^	26.9 ± 1.2	28.2 ± 1.7	27.6 ± 1.5
Total n−3 PUFA^4^	26.9 ± 1.3^b^	37.6 ± 1.9^a^	25.5 ± 3.2^b^	37.1 ± 2.4^b^	39.8 ± 1.3^a^	37.5 ± 1.6^b^
C16 PUFA	2.3 ± 0.2	2.0 ± 0.1	2.0 ± 0.1	2.5 ± 0.2^a^	2.6 ± 0.1^a^	2.3 ± 0.1^b^
Total PUFA	38.5 ± 1.9^b^	43.8 ± 1.7^a^	36.4 ± 2.2^b^	16.5 ± 2.9	45.7 ± 1.0	46.0 ± 1.6
DHA/EPA	2.1 ± 0.0^b^	2.6 ± 0.3^a^	2.9 ± 0.7^a^	4.5 ± 0.3^a^	3.5 ± 0.2^b^	4.5 ± 0.2^a^
n−3/n−6	2.3 ± 0.0^b^	6.0 ± 0.5^a^	2.4 ± 0.5^b^	4.0 ± 0.1^b^	6.7 ± 0.5^a^	4.4 ± 0.2^b^
Unknown	3.5 ± 0.2^c^	4.6 ± 0.3^b^	6.9 ± 0.3^a^	3.7 ± 0.2^b^	5.8 ± 0.4^a^	3.7 ± 0.4^b^

Results are means ± SD (*n* = 2). An SD of 0.0 implies an SD of < 0.05. Mean values within the same dietary trial bearing different superscript letters are significantly different (*P* < 0.05). ^1^Totals include 15:0, 20:0, 22:0, and 24:0. ^2^Totals include 16:1n−9, 18:1n−11, 20:1n−7, 22:1 isomers, and 24:1. ^3^Totals include 18:3n−6, 20:2n−6, and 22:4n−6. ^4^Totals include 20:3n−3 and 22:3n−3

*DHA* docosahexaenoic acid, *EPA* eicosapentaenoic acid, *MGK* Magokoro, *MUFA* monounsaturated fatty acid, *PUFA* polyunsaturated fatty acid, *SFA* saturated fatty acid, *15KO* diet containing 15% total lipid as krill oil, *20KO* diet containing 20% total lipid as krill oil, *15KORO* diet containing 15% total lipid as 50% krill oil and 50% rapeseed oil

No differences were observed in muscle lipid content among fish fed the different diets in either trial A or trial B (Tables 5 and 6). In trial A, fish fed 15KO and 20KO had the highest DHA and total n−3 LC-PUFA contents, while no differences were found in muscle total PUFA levels and, similarly, no differences were found in total saturated fatty acids whereas fish fed diet MGK had the highest monoene and n−6 PUFA contents (Table 5). In trial B, proportions of n−3 PUFA and saturates were highest in muscle of fish fed diet 15KO, whereas muscle of these fish showed the lowest percentages of MUFA and n−6 PUFA (Table 6). Fish fed MGK had the highest content of EPA although no differences were observed in DHA levels among fish fed the different diets in trial B.

### Gene transcript abundance in liver

#### Transcript abundance of lipid metabolism genes in liver of juvenile ABT

**Fig. 1:**
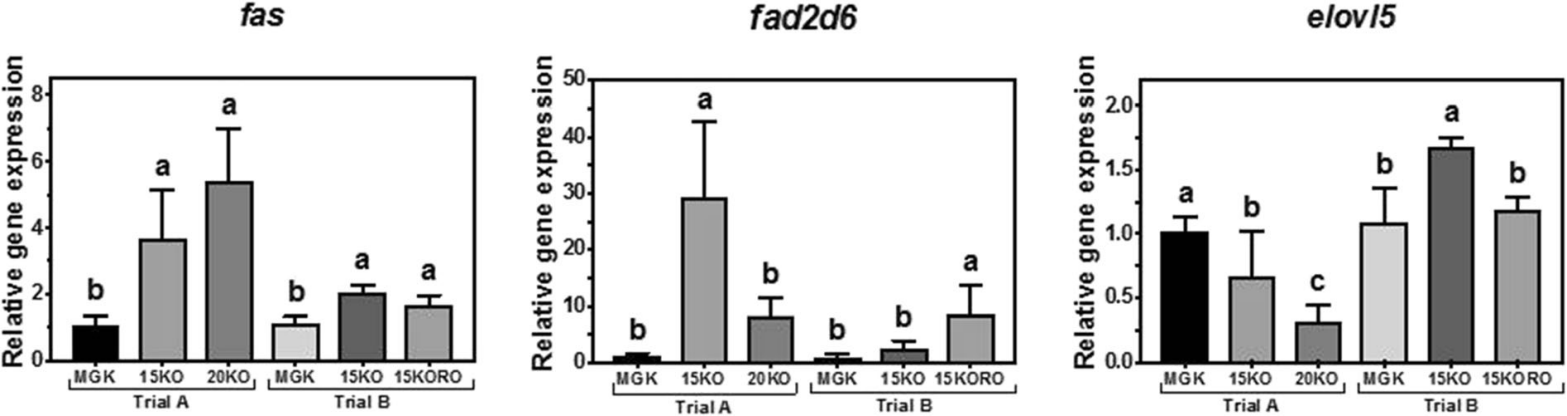
Nutritional regulation of fatty acid synthase (*fas*), delta-6 fatty acyl desaturase (*fads2d6*) and fatty acyl elongase 5 (*elovl5*) gene transcription in liver of Atlantic bluefin tuna (*Thunnus thynnus* L.) juveniles fed in dietary trial A, **a** reference diet Magokoro® (MGK), **b** 15% lipid diet based on krill oil (15KO), and **c** 20% lipid diet based on krill oil (20KO); and in dietary trial B, **a** Magokoro® (MGK) reference, **b** 15% lipid diet based on krill oil (15KO), and **c** 15% lipid diet based on krill oil and rapeseed oil 1:1; *v*/v (15KORO). Values are normalized expression ratios (to MGK reference diets in trials A and B) and are means ± SD of 6 individuals (*n* = 6). Values with different superscript letters are significantly different (one-way ANOVA and Tukey test; *P* < 0.05)

The transcript abundance of *fas* in liver of ABT juveniles was significantly higher in fish fed the experimental diets compared to fish fed the reference diet in both trials, although the response was quantitatively lower in trial B (Fig. 1). Concerning LC-PUFA biosynthesis genes, in trial A, *fads2d6* transcript abundance was significantly higher in fish fed diet 15KO whereas no differences were observed between fish fed the other two diets (Fig. 1). In contrast, *elovl5* transcript abundance was highest in fish fed diet MGK, with abundance in fish fed diet 20KO (with the highest level of dietary DHA and total n−3 PUFA) significantly lower than that in fish fed diet 15KO. On the other hand, in trial B, *fads2d6* transcript abundance was significantly higher in fish fed 15KORO (lowest dietary DHA and total n−3 PUFA contents), whereas no significant differences in abundance were observed between fish fed diet 15KO compared to fish fed MGK (Fig. 1). Transcript abundance of *elovl5* was highest in fish fed diet 15KO (highest DHA and total n−3 PUFA contents), but no significant differences in abundance of transcripts in this gene were observed between fish fed 15KORO and MGK (Fig. 1).

**Fig. 2:**
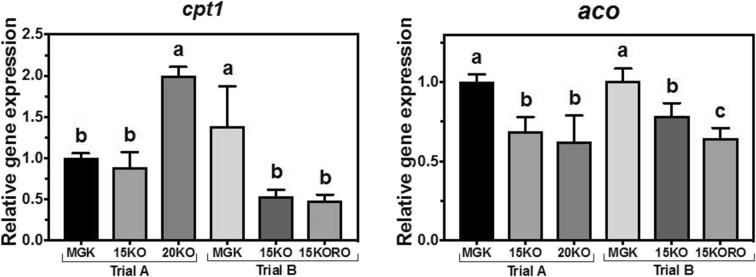
Nutritional regulation of carnitine palmitoyl transferase I (*cptI*) and acyl coA oxidase (*aco*) gene transcription in liver of Atlantic bluefin tuna (*Thunnus thynnus* L.) juveniles fed in dietary trial A, **a** reference diet Magokoro® (MGK), **b** 15% lipid diet based on krill oil (15KO), and **c** 20% lipid diet based on krill oil (20KO); and in dietary trial B, **a** reference diet Magokoro® (MGK), **b** 15% lipid diet based on krill oil (15KO), and **c** 15% lipid diet based on krill oil and rapeseed oil 1:1; *v*/*v* (15KORO). Values are normalized expression ratios (to MGK reference diets in trials A and B) and are means ± SD of 6 individuals (*n* = 6). Values with different superscript letters are significantly different (one-way ANOVA and Tukey test; *P* < 0.05)

In trial A, hepatic *cpt1* transcript abundance was significantly higher in fish fed diet 20KO (highest dietary total lipid, DHA, and total n−3 PUFA), whereas abundance in fish fed diet 15KO was lower and not significantly different from that of fish fed MGK (Fig. 2). In contrast, in trial B, the pattern was inverted, with hepatic transcript abundance of *cpt1* in fish fed diets 15KO and 15KORO similar and lower compared to fish fed the MGK diet. Nutritional regulation of the transcript abundance of peroxisomal *aco* showed a similar pattern in both trials. In trial A, fish fed diets 15KO and 20KO showed a significant downregulation compared to fish fed MGK, with no difference between the test diets. Similar results were shown in trial B (both test diets downregulated compared to MGK) but, in this case, the transcript abundance in fish fed diet 15KORO was significantly lower than that in fish fed diet 15KO (Fig. 2).

**Fig. 3:**
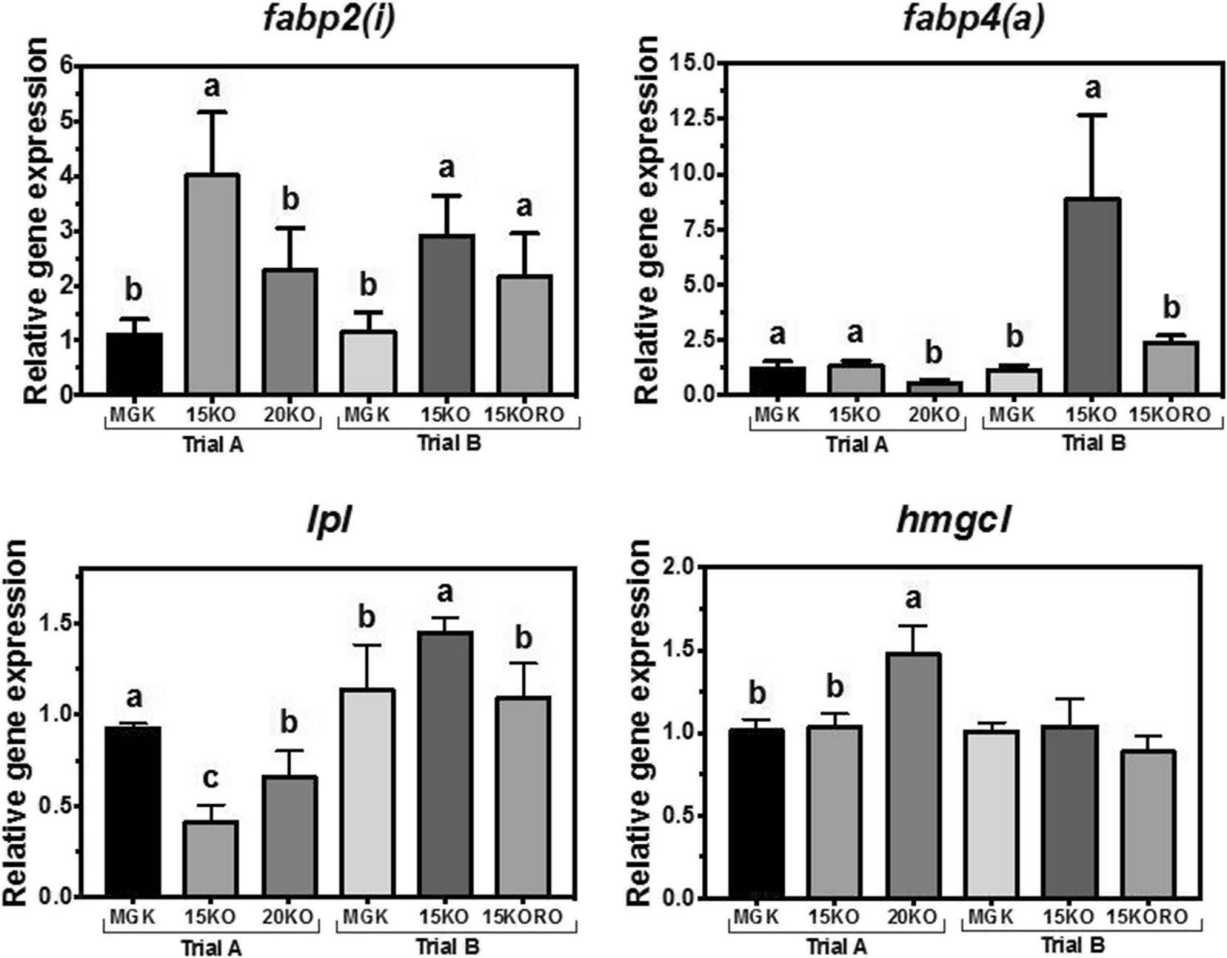
Nutritional regulation of fatty acid binding protein 2 and 4 (*fabp2* and *fabp4*, respectively), lipoprotein lipase (*lpl*), and 3-hydroxy-3-methylglutaryl-CoA lyase (*hmgcl*) gene transcription in liver of Atlantic bluefin tuna (*Thunnus thynnus* L.) juveniles fed in dietary trial A, **a** reference diet Magokoro® (MGK), **b** 15% lipid diet based on krill oil (15KO), and **c** 20% lipid diet based on krill oil (20KO); and in dietary trial B, **a** reference diet Magokoro® (MGK), **b** 15% lipid diet based on krill oil (15KO), and **c** 15% lipid diet based on krill oil and rapeseed oil 1:1; *v*/*v* (15KORO). Values are normalized expression ratios (to MGK reference diets in trials A and B) and are means ± SD of 6 individuals (*n* = 6). Values with different superscript letters are significantly different (one-way ANOVA and Tukey test; *P* < 0.05)

The nutritional regulation of liver genes related to fatty acid transport (*fabp2* and *fabp4*), lipid deposition (*lpl*), and mitochondrial lipid catabolism (*hmgcl*) is shown in Fig. 3. In trial A, *fabp2* transcript abundance was significantly higher in fish fed 15KO with no differences between fish fed MGK and 20KO. In trial B, a similar pattern was shown but, in this case, the transcript expression of *fabp2* in liver of fish fed both test diets (15KO and 15KORO) was significantly higher compared to fish fed MGK. The transcript abundance of *fabp4* in trial A was significantly lower in liver of fish fed diet 20KO compared to abundance in fish fed diets MGK and 15KO, which were identical. In trial B, the transcript abundance of *fabp4* was significantly higher in fish fed 15KO. The transcript abundance of *lpl* was lower in fish fed 15KO and 20KO compared to fish fed the MGK diet in trial A but, in contrast, *lpl* transcript abundance in fish fed 15KO was higher than those fed the other two diets in trial B. The hepatic transcript abundance of *hmgcl* in trial A was higher in fish fed diet 20KO, whereas in trial B, no significant differences were found among diets for the abundance of transcripts of this gene.

#### Transcript abundance of transcription factor genes in liver of juvenile ABT

**Fig. 4:**
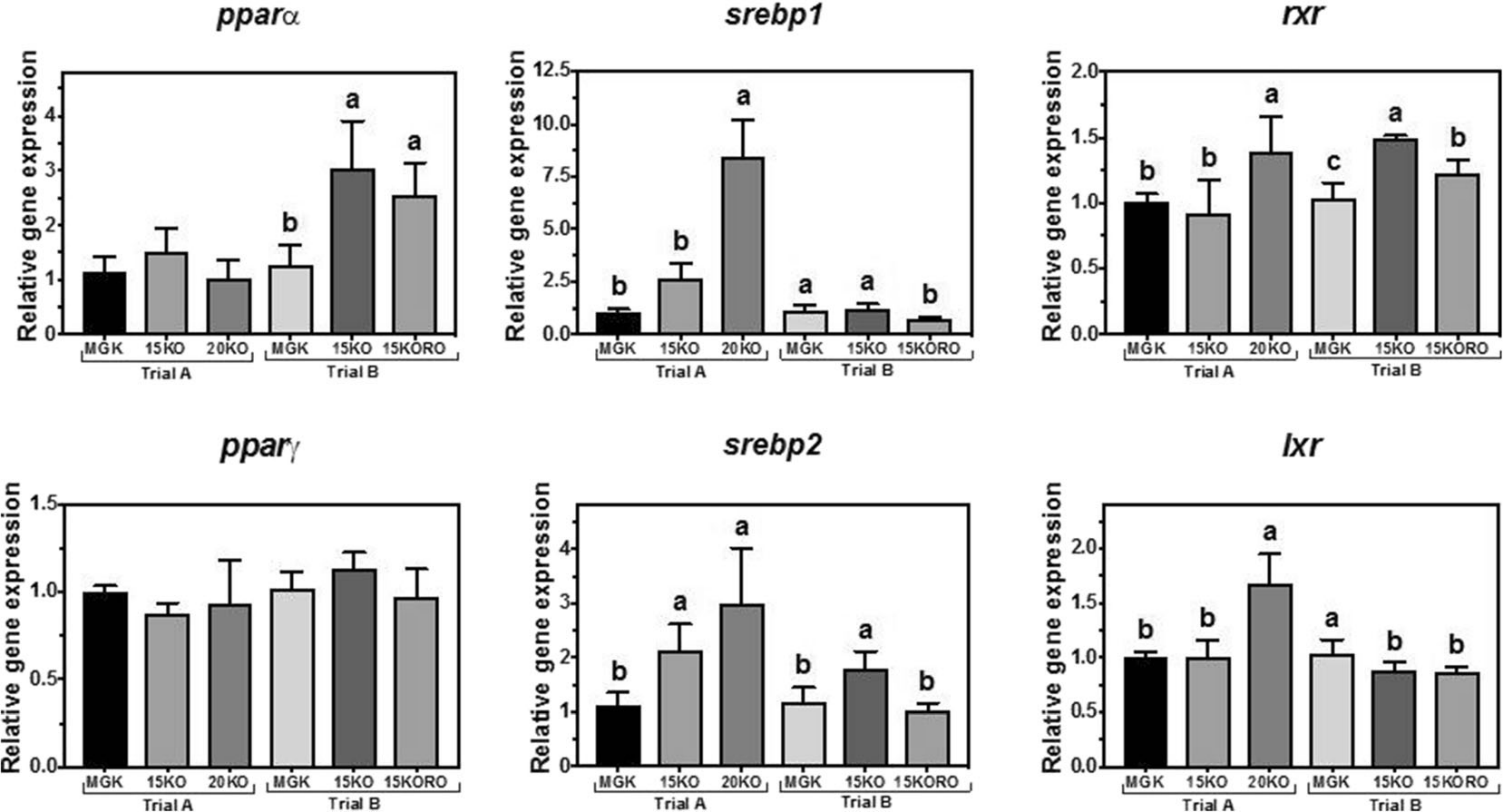
Nutritional regulation of peroxisome proliferator-activated receptor alpha (*pparα*)*,* gamma (*pparγ*)*,* sterol regulatory element-binding protein 1 and 2 (*srebp1*and *srebp2* respectively), retinoid X receptor (*rxr*), and liver X receptor (*lxr*) gene transcription in liver of Atlantic bluefin tuna (*Thunnus thynnus* L.) juveniles fed in dietary trial A, **a** reference diet Magokoro® (MGK), **b** 15% lipid diet based on krill oil (15KO), and **c** 20% lipid diet based on krill oil (20KO); and in dietary trial B, **a** reference diet Magokoro® (MGK), **b** 15% lipid diet based on krill oil (15KO), and **c** 15% lipid diet based on krill oil and rapeseed oil 1:1; *v*/*v* (15KORO). Values are normalized expression ratios (to MGK reference diets in trials A and B) and are means ± SD of 6 individuals (*n* = 6). Values with different superscript letters are significantly different (one-way ANOVA and Tukey test; *P* < 0.05)

In trial A, *pparα* did not show any significant differences in the relative transcript abundance among dietary treatments while in trial B, *pparα* transcript abundance in fish fed both test treatments (15KO and 15KORO) was significantly higher compared to fish fed MGK (Fig. 4). In contrast, abundance of *pparγ* gene transcript did not show any differences among the dietary treatments in either trial A or B. Abundance of *srebp1* gene transcripts was highest in fish fed 20KO in trial A, and in trial B it was lowest in fish fed 15KORO (Fig. 4). In trial A, *srebp2* showed a pattern of transcript abundance similar to that of *srebp1* and, in trial B, transcript abundance of *srebp2* was highest in liver of fish fed diet 15KO. In trial A, *rxr* and *lxr* showed similar patterns of transcript abundance, highest in fish fed diet 20KO but, in Trial B, *rxr* transcript abundance was higher in fish fed both test diets compared to MGK, whereas *lxr* was lower in fish fed 15KO and 15KORO.

#### Abundance of transcripts of antioxidant defense enzyme genes

**Fig. 5:**
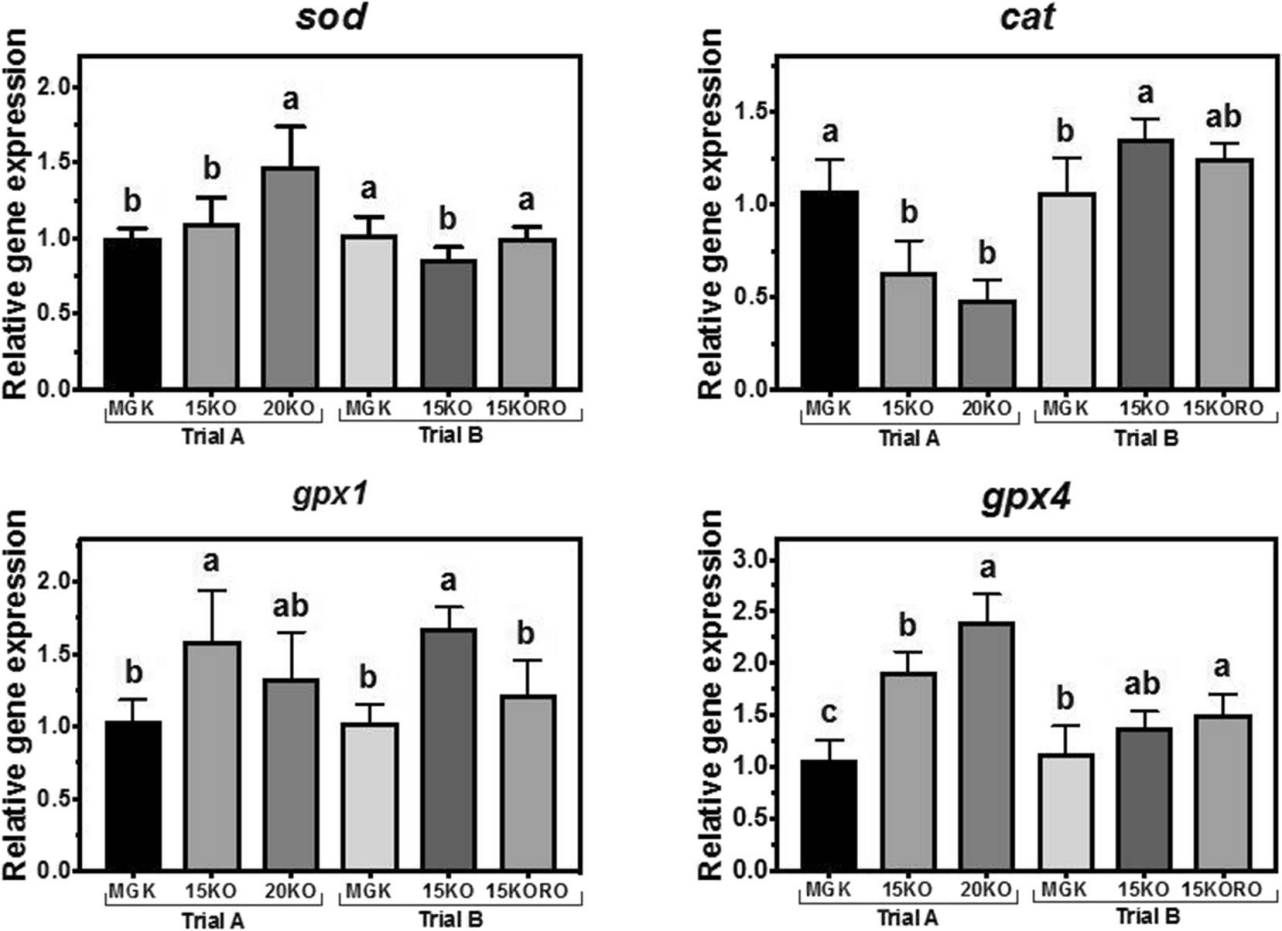
Nutritional regulation of superoxide dismutase (*sod*)*,* catalase (*cat*), and glutathione peroxidase 1 and 4 (*gpx1* and *gpx4*, respectively) gene transcription in liver of Atlantic bluefin tuna (*Thunnus thynnus* L.) juveniles fed in dietary trial A, **a** reference diet Magokoro® (MGK), **b** 15% lipid diet based on krill oil (15KO), and **c** 20% lipid diet based on krill oil (20KO); and in dietary trial B, **a** reference diet Magokoro® (MGK), **b** 15% lipid diet based on krill oil (15KO), and **c** 15% lipid diet based on krill oil and rapeseed oil 1:1; *v*/*v* (15KORO). Values are normalized expression ratios (to MGK reference diets in trials A and B) and are means ± SD of 6 individuals (*n* = 6). Values with different superscript letters are significantly different (one-way ANOVA and Tukey test; *P* < 0.05)

In trial A, the abundance of *sod* gene transcripts was higher in fish fed diet 20KO compared to MGK-fed fish while in trial B, *sod* in fish fed 15KO was lower than in fish fed MGK (Fig. 5). The transcript abundance of *cat* was downregulated in response to the test diets in trial A, whereas in trial B fish fed both test diets showed an upregulation of this gene. The patterns of *gpx1* and *gpx4* transcripts abundance were similar in both trials with *gpx1* abundance being highest in fish fed 15KO and abundance of *gpx4* being highest in 20KO and 15KORO (Fig. 5).

#### Correlations in transcript abundance

Some correlations could be established between the transcript abundance of the evaluated genes and other parameters, some of them being common in both trials (Table 7). For example, a strong negative correlation was found between *fas* gene transcript abundance and liver lipid content, whereas a positive correlation was found between this gene and liver DHA content in both trials. The abundance of transcripts of *gpx1* and *gpx4* was also positively correlated with dietary taurine and Se contents, and hepatic contents of DHA and n−3 PUFA, in both trials. Positive correlations were also found between *srebp1* transcript abundance and dietary DHA, dietary n−3 PUFA, *fas*, and *lxr* abundance levels in both trials. The transcript abundance of *lxr* was positively correlated with dietary lipid content in both trials whereas, in contrast, it was positively correlated with *fas* gene transcript abundance in trial A, but negatively correlated in trial B.

**Table 7:**
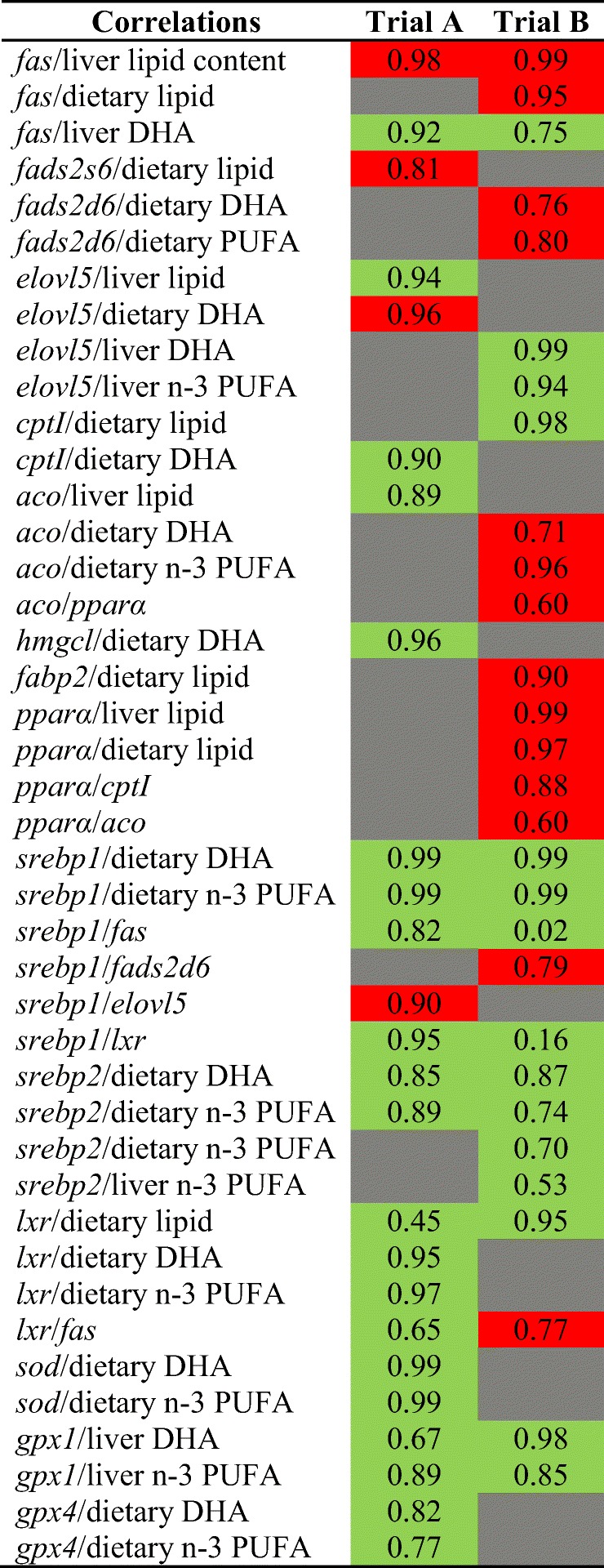
Correlations established between the expression of the evaluated genes and other parameters.

Green cells indicate positive correlation; red cells represent negative correlation whereas gray cells indicate that no significant correlation was found. The *R*^2^ value is indicated within each cell

*Aco* acyl coA oxidase, *cptI* carnitine palmitoyl transferase I, *elovl5* fatty acyl elongase 5, *fabp2* fatty acid binding protein 2 (intestinal), *fads2d6* delta-6 fatty acyl desaturase, *fas* fatty acid synthase, *gpx1* glutathione peroxidase 1, *gpx4* glutathione peroxidase 4, *hmgcl* 3-hydroxy-3-methylglutaryl-CoA lyase, *lxr* liver X receptor, *pparα* peroxisome proliferator-activated receptor alpha, *sod* superoxide dismutase, *srebp1* sterol regulatory element-binding protein 1, *srebp2* sterol regulatory element-binding protein 2

## Discussion

Lipid and protein metabolic pathways dominate intermediary metabolism in carnivorous fish, such as ABT and PBT, as carbohydrate is only a minor component in natural diets in the wild (Tocher 2003; Mourente and Tocher 2003, 2009). The optimum dietary protein, lipid, and carbohydrate contents have been determined for PBT juveniles (Biswas 2010; Kenji 2012), and it has been suggested that protein and lipid imbalances in formulated diets can result in lower growth performance (Biswas et al. 2009). Results from the trials in the present study indicated that diets with relatively high protein to lipid ratios of between 3 and 4 could support good growth of juvenile ABT.

Availability of appropriate tanks meant that the present study was run as two separate trials rather than one large trial and, serendipitously, this has provided some important insight. While fish more than doubled their weight in both trials, a difference was observed regarding performance of ABT in the two trials, as fish fed the commercial MGK diet showed higher growth than fish fed the experimental feeds (cf. diet 15KO, which was in both trials) in trial A and lower growth in trial B. While MGK was not specifically formulated for ABT, it was included in this study as the most appropriate reference diet to provide a benchmark for the performance of the experimental feeds. As such, it is therefore important to acknowledge that it is not a control diet and varied from the experimental feeds in terms of micronutrient contents (see Table 2). In general, in the present study, values measured for growth performance, such as weight gain and SGR, were numerically lower than those observed in trials with PBT juveniles (Biswas 2010; Biswas et al. 2009; Kenji 2012), but similar to those found by Biswas et al. (2011). However, species differences alone cannot explain the differential in performance observed between the two trials. To explain this, it should be noted that the fish used in the two trials were derived from batches of eggs produced at different times in the season from a stock of captive wild broodstock, which could highlight the importance of the genetic background or nutritional status of the broodstock. Indeed, no significant differences were found in relation to feed utilization variables in the trials, which suggests that differences in growth may not be directly or solely related to the feeds themselves. In contrast, it is highly likely that genetic background of the broodstock influenced the data obtained. The difference between the two batches reflects the normal biological variation between individuals and, if different males and, especially, female broodstock contributed to the fertilized eggs in each batch, as is highly likely, the batches would be consequently different. If many different males and females contributed to each batch, then the variation between batches may be less. Therefore, the data suggest that a limited number of individuals contributed to the batches of fertilized eggs and possibly only one female could have spawned each batch of eggs.

Diet had no significant impact on survival partly due to high variability among treatments. However, it is well known, as reported in dietary trials performed with PBT juveniles, that a major part of the mortality at this stage is associated with factors related to stress responses to external stimuli (light, noise, etc.) rather than to dietary deficiencies themselves, in most cases resulting in collisions with the tank wall and often death of the fish (Biswas 2010; Biswas et al. 2009; Cho et al. 2016).

In the present study, neither increased dietary lipid level nor dietary RO increased liver or muscle lipid content. Hepatic lipid deposition is a result of a balance between fatty acid oxidation, synthesis, and transport but, generally, dietary fatty acid composition is reflected in the fish body (Turchini et al. 2009). In the present study, liver fatty acid profiles generally reflected those of diets, as shown previously in other fish studies (Betancor et al. 2014a; Araújo et al. 2017). This clearly indicated that, despite being relatively short, the feeding trials were sufficiently long in these very fast-growing animals to result in the changes in biochemical composition that would be expected in fish that showed a more than doubling of weight. However, levels of fatty acids such as 16:0, 18:0, ARA, and DHA presented slightly higher levels in liver compared to diet, with this more accentuated in liver of fish fed the diet with higher lipid content (20KO in trial A). In contrast, other fatty acids such as 16:1n−7, 18:1n−9, total monoenes, LNA, 18:4n−3, 20:4n−3, EPA, and 22:5n−3 were present in liver at lower percentages than in diet. In addition, liver of fish fed the diet containing vegetable oil (15KORO) showed lower levels of n−3 LC-PUFA, and higher levels of 18:1n−9, LA, and LNA, compared to fish fed the other diets. Higher levels of fatty acids in liver compared to feeds are probably due more to selective deposition than to biosynthesis, whereas lower levels in liver may reflect utilization for energy. On the other hand, ABT can be recognized as a lean fish at this fast-growing juvenile stage, and this was reflected in the muscle lipid content, which, in contrast to liver, was very constant and low (0.8–0.9%). Similar to several previous studies in other fish species (Bell et al. 2002; Betancor et al. 2014a), the fatty acid profile of juvenile ABT muscle reflected dietary intake and, thus, fish fed the diets containing KO as the sole added lipid source accumulated high contents of DHA in muscle compared to fish fed the commercial control or KORO feeds. Thus, while using more sustainable feeds containing terrestrial plant ingredients did not impact ABT growth, it can reduce the contents of the health-promoting omega-3 LC-PUFA in their flesh, reducing, in turn, the nutritional value for the consumer.

Generally, upregulation of *fads2d6* expression is observed in fish fed low dietary levels of n−3 LC-PUFA, whereas high dietary levels have been associated with reduced expression (Morais et al. 2012; Betancor et al. 2015a). In the present study, upregulation of *fads2d6* expression was observed in juvenile ABT fed the test diets in relation to the reference diet (primarily in diet 15KO in trial A and diet 15KORO in trial B), while no clear pattern was observed in the expression of *elovl5*, which was downregulated with test diets in trial A and slightly upregulated in 15KO-fed fish in trial B. In a previous trial with ABT larvae (Betancor et al. 2017a), no nutritional regulation of *fads2d6* and *elovl5* was observed and the authors hypothesized that this suggested all the diets provided sufficient n−3 LC-PUFA to satisfy minimum requirements and thus expression of the genes did not differ. Although differences in the DHA contents of the diets used in the previous trial (13.5–28.3 μg mg^−1^) and the present trial (15.5–29.5 μg mg^−1^) were similar, the observed difference in the regulation of the LC-PUFA genes between the studies may indicate an effect of life stage, with different requirements and/or basal biosynthetic activity/expression in larvae versus juveniles. On the other hand, transcript abundance has not always been shown to correlate with enzyme activity or protein abundance (Maier et al. 2009) that might explain the lack of consistency between the two experiments.

The transcriptional mechanisms that control the synthesis, storage, release, uptake, and oxidation of fatty acids, as the main participants in energy homeostasis, are poorly understood in fish and may vary with fish species (Dong et al. 2017). In fish, as in mammals, two transcription factors (*srebp1* and *ppar*) are implicated in fatty acid biosynthesis and catabolism, respectively (Desvergne et al. 2006; Dong et al. 2017), and their expression can be regulated by dietary fatty acids (Zheng et al. 2009; Minghetti et al. 2011; Dong et al. 2015). Moreover, high dietary levels of n−3 LC-PUFA, including DHA, can act as ligands for transcription factors such as *pparα* and *srebp1*, downregulating the biosynthesis of LC-PUFA (Worgall et al. 1998; Hihi et al. 2002; Cunha et al. 2013; Peng et al. 2014) and regulating the expression of their target genes such as *fas*, *cptI*, *aco*, or *lpl*. In the present study, *srebp1* transcript abundance was strongly correlated with those of *fas* and *lxr* in both trials. In mammals, it has been shown that *srebp1* expression is stimulated by the expression of *lxr* (Desvergne et al. 2006), and the results in the present study are also consistent with this. However, previous studies in several fish species showed that *srebp1* expression was upregulated when feeds contained only low levels of EPA and DHA (Geay et al. 2011; Morais et al. 2011; Betancor et al. 2014a; Limtipsuntorn et al. 2014; Peng et al. 2014). In contrast, in the present study, fish fed the diets with the highest lipid and n−3 LC-PUFA contents showed upregulation of both *lxr* and *srebp1*, and *srebp1* expression was positively correlated to dietary DHA and total n−3 PUFA in both trials. It should be noted that the expression of *srebp1*, *lxr*, and *fas* showed strong positive correlations with dietary DHA and total n−3 PUFA, with weaker responses in trial B due to the dilution of these components as a result of the inclusion of RO in diet 15KORO. Given the consistency of these results, the up- and downregulation of *srebp1* and *lxr* transcript abundance, respectively, in liver of ABT juveniles might be driven by the decreased/increased lipid accumulation through a feedback mechanism, possibly at post-transcriptional and/or translational levels. On the other hand, *lxr* acts to regulate the formation of bile acids from cholesterol in mammals (Desvergne et al. 2006) and, in the present study, *lxr* expression was positively correlated to dietary lipid content, which may be associated with increased synthesis of bile acids to emulsify dietary lipids. Indeed, *srebp2* regulates the expression of genes involved in cholesterol synthesis (Jeon and Osborne 2012; Carmona-Antoñanzas et al. 2014) and is upregulated in response to reduced cholesterol (Minghetti et al. 2011; Carmona-Antoñanzas et al. 2014). In trial A, a similar trend of expression was observed between *lxr* and *srebp2*.

Fatty acids absorbed by liver supply energy through β-oxidation with *pparα* stimulating hepatic β-oxidation by inducing expression of a set of target genes that participate in many, if not all, aspects of lipid catabolism including key genes *cpt1* and *aco* (Desvergne et al. 2006; Goto et al. 2011). In trial B, the expression of *pparα* was negatively correlated to those of *cptI* and *aco* in response to the test diets, exerting a downregulating effect in fish fed these diets. This may indicate that most dietary lipid was being used by ABT juveniles for membrane biosynthesis during this fast-growing period, with less lipid used for energy production via β-oxidation, suggesting that most energy was being supplied by the high dietary protein. The transcription factor, *pparγ*, plays important roles in lipogenesis, lipid storage and adipogenesis, and osteogenesis (Nedergaard et al. 2005; Ji et al. 2011; Agawa et al. 2012). However, *pparγ* showed no regulation in the present study, with no significant differences among dietary treatments in either trial. Similar results were obtained in a 7-day dietary trial with juvenile PBT using test diets formulated with different oil sources, with no differences in *pparγ* gene expression reported (Agawa et al. 2012). This might indicate that during fast growth of tuna juveniles, increase in biomass prevails over lipid storage, with most of the available dietary resources utilized for anabolic processes and growth.

Fatty acid binding proteins (FABP) are involved in fatty acid uptake, transport, and metabolism (Glatz and van der Vusse 1996), serving as carriers of saturated and unsaturated long-chain fatty acids, eicosanoids, and other hydrophobic ligands to effector molecules in the cytosol and nucleus (Esteves et al. 2015). In the present study, *fabp2* gene expression was higher in ABT fed the test diets containing 15% lipid (15KO and 15KORO) than in the fish fed higher dietary lipid (20KO). In a previous trial, *fabp2* expression was higher when larvae were fed live prey containing 11% lipid and lower when fed prey with 5.6% lipid, which could indicate that lipid content regulates expression of this gene in larvae, rather than dietary fatty acid composition (Betancor et al. 2017a). However, Atlantic cod and gilthead sea bream juveniles fed diets containing vegetable oil showed upregulation in the expression of this gene (Lilleeng et al. 2007; Betancor et al. 2016). On the other hand, *fabp4* gene expression showed a different pattern, being lower in fish fed diet 20KO in trial A and higher in fish fed 15KO in trial B. In previous studies in fish, a correlation between β-oxidation capacity and expression levels of *fabps* has been suggested (Londraville and Sidell 1996; Torstensen et al. 2009) but, in the present study, no strong correlations between the expression of *fabp*s genes and the expression of genes or transcription factors related to β-oxidation were observed. The differing expression patterns of different lipid metabolism genes observed between the two trials could be a further influence of genetic background based on the fact that the ABT juveniles came from different spawnings as described above. However, it could also reflect daily rhythms of expression of lipid metabolism genes as has been shown in several teleost species (Betancor et al. 2014b; Hernández-Pérez et al. 2015; Paredes et al. 2015).

In a previous study, *lpl* was found to be highly expressed in muscle and liver of adult ABT (Betancor et al. 2017b). The *lpl* gene encodes a lipolytic enzyme responsible for lipid uptake in adipocytes and represents an early marker of adipocyte differentiation. However, studies on the nutritional regulation of *lpl* expression in fish are scarce. In red sea bream, both feeding conditions and dietary lipid and fatty acid levels were shown to alter *lpl* expression in liver, with lower expression in fish fed low lipid than in fish fed high lipid (Liang et al. 2002a). In the present study, the expression of *lpl* in liver of ABT juveniles was low and showed no clear response to diet, similar to previous studies where ABT larvae fed different live prey showed variable *lpl* expression (Betancor et al. 2017a, b). In contrast, *hmgcl* expression was upregulated in ABT juveniles fed higher lipid (20KO), while previous studies in larvae did not show any dietary regulation of this gene (Betancor et al. 2017a, b) although increased expression of this enzyme has been associated with enhanced fatty acid oxidation in rat liver (Ide et al. 2009). Therefore, in the present study, the upregulation of *hmgcl* in fish fed the higher lipid content could be related to enhanced oxidation of lipids, consistent with the regulation of *cpt1*.

To maintain health and prevent oxidation-induced pathology and mortalities, there must be effective antioxidant systems operating in fish. Enzyme components of antioxidant defense include catalase (CAT), superoxide dismutase (SOD), and glutathione peroxidases (GPX), as well as associated enzymes such as glutathione-*S*-transferase (GST) and glutathione reductase (GR). Lipid peroxidation via ROS is quantitatively the main peroxidative process in mitochondria, damaging the membrane lipids. In fish, changes in dietary fatty acid composition can modify mitochondrial membrane composition and alter organelle function leading to an imbalance in oxidative status by affecting the ability to maintain the structural homeostasis of membranes (Betancor et al. 2015b). In the present study, dietary antioxidant protection in terms of antioxidant vitamins, Se, and taurine contents varied between the reference and test diets. Vitamins E and C contents were around 5- and 2-fold higher in MGK than in the test diets although vitamin C levels in the test diets could be considered optimal since their values were well above those recommended by Biswas et al. (2013) for juvenile PBT (454 mg kg^−1^ diet). In contrast, Se and taurine concentrations were higher in the test feeds than in the reference diet. Taurine has an antioxidant role among other roles (NRC 2011; Salze and Davis 2015) and Se, as a component of the glutathione peroxidase enzyme family, functions as a biological antioxidant (NRC 2011). As stated above, the expression of *gpx1* and *gpx4* were positively correlated with dietary taurine and Se contents in both trials. It could be expected that, if contents of vitamins E and C in the test diets were low or insufficient, the liver antioxidant system enzymes of ABT juveniles may compensate to protect from oxidative stress. This could be the situation in some cases where the expression of the genes *sod*, *cat*, *gpx1*, and *gpx4* were upregulated in fish fed the test diets (with lower antioxidant vitamins) in comparison to fish fed the reference diet.

In conclusion, the present study suggested that ABT juveniles can be on grown on inert extruded dry feeds with good fish growth and accumulation of the health-promoting fatty acid DHA. Furthermore, a blend of vegetable and krill oils could be used as the dietary lipid source up to a dietary lipid level of 15% without affecting fish performance. The expression of lipid metabolism genes in ABT liver showed a different response to dietary lipid level/fatty acid profile consistent with previous data indicating limited n−3 LC-PUFA biosynthetic capability in ABT. However, gene expression sometimes differed between the two trials, which may highlight that genetic background of different batches of ABT juveniles could affect the regulation of metabolic gene expression and thus be a factor in weaning success. The expression of antioxidant enzymes was also altered by diet, related to dietary contents of antioxidant nutrients. Further studies are required in order to fully elucidate lipid and fatty acid requirements of this iconic species in relation to dietary sources and production costs.

## Electronic supplementary material

ESM 1 (DOCX 21 kb)

## Abbreviations

ABT: Atlantic bluefin tuna
*aco*: Acyl coA oxidase
ARA: Arachidonic acid (20:4n−6)
*cat*: Catalase
*cpt1*: Carnitine palmitoyl transferase I
dah: Days after hatch
DHA: Docosahexaenoic acid (22:6n−3)
EFA: Essential fatty acid
EPA: Eicosapentaenoic acid (20:5n−3)
*elovl5*: Fatty acyl elongase 5
*fabp2*: Fatty acid binding protein 2 (intestinal)
*fabp4*: Fatty acid binding protein 4 (adipocyte)
*fabp7*: Fatty acid binding protein 7 (brain-type)
*fads2d6*: Delta-6 fatty acyl desaturase
*fas*: Fatty acid synthase
*gpx1*: Glutathione peroxidase 1
*gpx4*: Glutathione peroxidase 4
*hmgcl*: 3-Hydroxy-3-methylglutaryl-CoA lyase
LA: Linoleic acid (18:2n−6)
LC-PUFA: Long-chain polyunsaturated fatty acid
LNA: α-Linolenic acid (18:3n−3)
*lpl*: Lipoprotein lipase
*lxr*: Liver X receptor
MUFA: Monounsaturated fatty acid
*pparα*: Peroxisome proliferator-activated receptor alpha
*pparγ*: Peroxisome proliferator-activated receptor gamma
PUFA: Polyunsaturated fatty acid
*rxr*: Retinoid X receptor
SFA: Saturated fatty acid
*sod*: Superoxide dismutase
*srebp1*: Sterol regulatory element-binding protein 1
*srebp2*: Sterol regulatory element-binding protein 2
