# Supplementary material for: Performance, feed utilization, and hepatic metabolic response of weaned juvenile Atlantic bluefin tuna (Thunnus thynnus L.): effects of dietary lipid level and source
Source: Fish Physiol Biochem. 2018 Nov 23;45(2):697–718. doi: 10.1007/s10695-018-0587-9 (PMC6500510; doi:10.1007/s10695-018-0587-9)
Supplement: Supplementary file 1 — (DOCX 21 kb) [file 10695_2018_587_MOESM1_ESM.docx]

**Table S1 (Supplementary).** Rearing conditions for feeding trials of Atlantic bluefin tuna (*Thunnus thynnus* L.) juveniles.

|  | **Trial A** | **Trial B** |
| --- | --- | --- |
| Initial age of fish (days after hatching) | 41 dah | 41 dah |
| Experimental period | 10 days | 10 days |
| Tank volume (m^3^) | 5 | 5 |
| Number of fish (ind/tank) | 40 | 46 |
| Feeding amount (g/tank/day) | “ad libitum” | “ad libitum” |
| Water temperature (°C) | 29.3 ± 1.0 | 28.6 ± 1.1 |
| Dissolved oxygen (mg/l) | 5.9 ± 0.5 | 5.6 ± 0.3 |
| Photoperiod (L:D) | 14:10 | 14:10 |
| Light intensity (lux) | 1000 | 1000 |
| Exchange rate of sea water (%tank volume/day) | 300-500 % | 250-500 % |

**Table S2 (Supplementary)**. Sequence, annealing temperature (Tm) and size of the fragment produced by the primer pairs used for quantitative PCR (qPCR).

| Name | Sequence (5’-3’) | Amplicon size (bp) | Tm°C |
| --- | --- | --- | --- |
| *elovl5* | F: CCACGCTAGCATGCTGAATA | 236 | 60 |
|  | R: ATGGCCATATGACTGCACAC |  |  |
| *fads2d6* | F: CCGTGCACTGTGTGAGAAAC | 152 | 60 |
|  | R: CAGTGTAAGCGATAAAATCAGCTG |  |  |
| *pparα* | F: TGGTCATGGAGGTGGAAGAC | 152 | 60 |
|  | R: ATGGATGACGAAAGGAGGGG |  |  |
| *pparγ* | F: ACCTGACCAACATGGACTAC | 118 | 60 |
|  | R: GAGAAAACAGGACTGTCAGC |  |  |
| *lxr* | F: CACACTGGATCCACAACAGC | 192 | 58 |
|  | R: ATCTCCTGCACCGACATGAT |  |  |
| *rxr* | F: TGAGGGAAAAGGTCTACGCA | 212 | 59 |
|  | R: TGTGATCTGATGTGGTGCCT |  |  |
| *srebp1* | F: CCAGCTACACATGACAGGGA | 153 | 59 |
|  | R: GCTTTGACCCTTAGAGCTGC |  |  |
| *srebp2* | F: AGATCCAGTGAGTCGTTGGT | 212 | 60 |
|  | R: CTACAGCCCCTTCTCCCTTC |  |  |
| *fabp2* | F: CGCAGCGAGAATTATGACAA | 244 | 55 |
|  | R: AGCATGTCACCCTCCATCTC |  |  |
| *fabp4* | F: ACTGCAATGACCGAAAGACC | 175 | 55 |
|  | R: CCTCCTTTCCGTAGGTCCTC |  |  |
| *fabp7* | F: CCTACACCTGATGACCGACA | 212 | 55 |
|  | R: GCTGGGATGATTTGCTCATT |  |  |
| *cptI* | F: TGGAGGCTGTCCACCAGTCA | 211 | 60 |
|  | R: TGCTGGAGATGTGGAAGTTG |  |  |
| *lpl* | F: CCGAAAGAACCGCTGCAATA | 212 | 59 |
|  | R: GATCCTCCTTCTCTCCGTGG |  |  |
| *fas* | F: ATACCGTGGCAATGTAACGC | 188 | 59 |
|  | R: GTGAGCTGTGGATGATGCTG |  |  |
| *aco* | F: AGCGCTATGACCAGGCTATT | 164 | 59 |
|  | R: GTACAGGGTTGGGAGGAACA |  |  |
| *hmgcl* | F: CGTGCCAACAGAGACGAAAA | 173 | 59 |
|  | R: GGGTGAGGACTGGGTAAGAC |  |  |
| *gpx1* | F: TGGAGAAAGTGGATGTGAACGG | 309 | 55 |
|  | R: GTGCTGTGGAAGCTGTATGATGG |  |  |
| *gpx4* | F: TGGGGAATAGCATCAAGTGG | 206 | 55 |
|  | R: CGAGAAAGGAGGGAAACAGG |  |  |
| *cat* | F: ATGGTGTGGGACTTCTGGAG |  | 60 |
|  | R: ATGAAACGGTAGCCATCAGG |  |  |
| *sod* | F: TCCCAGATCACCTACATGCC | 182 | 59 |
|  | R: CTGCGGAGAGTTGCTTGATC |  |  |
| *ef1a* | F: CCCCTGGACACAGAGACTTC | 119 | 60 |
|  | R: GCCGTTCTTGGAGATACCAG |  |  |
| *bactin* | F: ACCCACACAGTGCCCATCTA | 155 | 61 |
|  | R: TCACGCACGATTTCCCTCT |  |  |

*elovl5*, fatty acyl elongase 5; *fads2d6*, delta-6 fatty acyl desaturase; *pparα*, peroxisome proliferator-activated receptor alpha; *pparγ*, peroxisome proliferator-activated receptor gamma; *lxr*, liver X receptor; *rxr*, retinoid X receptor; *srebp1*, sterol regulatory element-binding protein 1; *srebp2*; sterol regulatory element-binding protein 2; *fabp2*, fatty acid binding protein 2 (intestinal); *fabp4*, fatty acid binding protein 4 (adipocyte); *fabp7*, fatty acid binding protein 7 (brain-type); *cptI*, carnitine palmitoyl transferase I; *lpl*, lipoprotein lipase; *fas*; fatty acid synthase; *aco*, acyl coA oxidase; *hmgcl*; 3-hydroxy-3-methylglutaryl-CoA lyase; *gpx1*, glutathione peroxidase 1: *gpx4*, glutathione peroxidase 4; *cat*, catalase; *sod*, superoxide dismutase; *ef1α*, elongation factor 1 alpha; *bactin*, beta actin.
